# Expressing a Z-disk nebulin fragment in nebulin-deficient mouse muscle: effects on muscle structure and function

**DOI:** 10.1186/s13395-019-0219-9

**Published:** 2020-01-28

**Authors:** Frank Li, Justin Kolb, Julie Crudele, Zaynab Hourani, John E. Smith, Jeffrey S. Chamberlain, Henk Granzier

**Affiliations:** 1 Department of Cellular and Molecular Medicine, University of Arizona, Tucson, AZ 85721 USA; 2 Department of Neurology, University of Washington, Seattle, WA 98109-8055 USA; 3 Medical Research Building, RM 325, 1656 E Mabel St, Tucson, AZ 85721 USA

**Keywords:** Nebulin, Nemaline myopathy, Gene therapy, AAV, Sarcomere, Thin filament, Muscle mechanics, Mouse models

## Abstract

**Background:**

Nebulin is a critical thin filament-binding protein that spans from the Z-disk of the skeletal muscle sarcomere to near the pointed end of the thin filament. Its massive size and actin-binding property allows it to provide the thin filaments with structural and regulatory support. When this protein is lost, nemaline myopathy occurs. Nemaline myopathy causes severe muscle weakness as well as structural defects on a sarcomeric level. There is no known cure for this disease.

**Methods:**

We studied whether sarcomeric structure and function can be improved by introducing nebulin’s Z-disk region into a nebulin-deficient mouse model (*Neb* cKO) through adeno-associated viral (AAV) vector therapy. Following this treatment, the structural and functional characteristics of both vehicle-treated and AAV-treated *Neb* cKO and control muscles were studied.

**Results:**

Intramuscular injection of this AAV construct resulted in a successful expression of the Z-disk fragment within the target muscles. This expression was significantly higher in *Neb* cKO mice than control mice. Analysis of protein expression revealed that the nebulin fragment was localized exclusively to the Z-disks and that *Neb* cKO expressed the nebulin fragment at levels comparable to the level of full-length nebulin in control mice. Additionally, the Z-disk fragment displaced full-length nebulin in control mice, resulting in nemaline rod body formation and a worsening of muscle function. *Neb* cKO mice experienced a slight functional benefit from the AAV treatment, with a small increase in force and fatigue resistance. Disease progression was also slowed as indicated by improved muscle structure and myosin isoform expression.

**Conclusions:**

This study reveals that nebulin fragments are well-received by nebulin-deficient mouse muscles and that limited functional benefits are achievable.

## Background

Nemaline myopathy is a rare congenital disease that disrupts the skeletal muscle sarcomeres and results in muscle weakness. This disease was originally identified by, and named for, the thread-like protein aggregates found in muscle biopsies [1, 2]. Patients diagnosed with nemaline myopathy exhibit a wide range of disease severities from manageable symptoms to severe disruptions to quality of life; while most patients exhibit a mild phenotype, in severe nemaline myopathy, muscle weakness can lead to respiratory failure and death. Despite studies into family genomes, no conclusive genotype-phenotype correlation has been found, though the disease has been separated into subtypes based on severity and onset [3–11]. The complexity of this disease has prevented the development of a reliable treatment, requiring individuals and caregivers to instead focus their attention on managing symptoms. There are now 13 genes known to contribute to the development of nemaline myopathy. Eight are associated with the thin filaments [3, 12–18], three are thought to participate in nebulin stabilization or turnover [19–21], and two are more peripherally associated with the development of nemaline myopathy [22, 23]. Of these genes, the thin filament regulatory protein nebulin is estimated to be responsible for approximately 50% of all observed cases of nemaline myopathy [3, 24].

Nebulin is one of the largest proteins in the human body [25]. It is a massive linear protein of ~ 700 kDa that extends from the Z-disks of the skeletal muscle sarcomeres out toward the pointed ends of the thin filaments [26]. In mice, its core structure is comprised of 206 homologous, repeat modules which each contain an SDxxYK actin-binding sequence [27–29]. These modules allow nebulin to associate closely with the actin thin filaments and contribute to its primary role as a thin filament length regulator. Additionally, 175 of these modules can also be grouped into 7-module super-repeats, which each contain a tropomyosin binding site that helps to integrate this protein into the thin filaments [27–30]. Outside of these modules, there is a glutamic acid-rich region at the N-terminus of the protein and two more unique domains at the C-terminus: the serine-rich region and the SH3 domain. While the glutamic acid-rich region remains unstudied, the serine-rich region and the SH3 domains are thought to contribute to the regulation of other sarcomeric proteins as well as the development of the Z-disk [31].

Due to nebulin’s contributions to thin filament length regulation, force production, and structural maintenance [31–41], several studies have focused on improving these aspects of the sarcomere through therapeutic interventions. Studies targeting troponin activation have reported increases in force production at submaximal stimulation frequencies [42, 43], but other attempts to improve muscle weight and function have shown that such therapeutic changes are difficult to achieve [44–46]. In this study, we sought to improve sarcomere structure and function by inserting a partial fragment of nebulin into the sarcomeres via an adeno-associated viral vector (AAV). Limitations in the packaging capacity prevented the design of a vector containing the full nebulin gene [47, 48]. However, current research has focused on the introduction of functional protein fragments or truncated proteins using this technology, reviewed in [49]. Here, we hypothesized that the expression of an exogenous nebulin fragment improves the structure and function of the sarcomere. Through a construct containing the Z-disk portion of nebulin, including the final super-repeat, we studied whether the Z-disk was targeted and if improvements in sarcomeric structure and function were attainable. After treating nebulin-deficient mice with the AAV for a 1-month period, we examined force production, sarcomeric structure, and expression of nebulin and accessory proteins.

## Materials and methods

### Generation of the Z-disk AAV construct

**Fig. 1:**
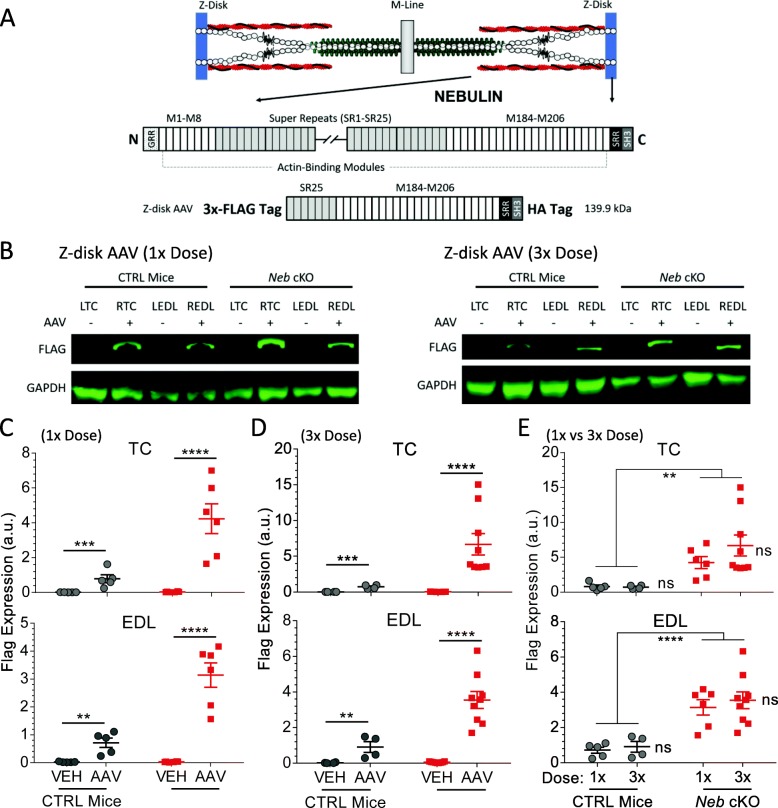
Expressing nebulin’s Z-disk region in control (CTRL) and nebulin-deficient (*Neb* cKO) mice. **a** Sarcomere highlighting nebulin wrapped around the thin filament (top), the structure of nebulin (middle), and nebulin’s Z-disk fragment (bottom). **b** Representative Western blot for FLAG tag signal (139.9 kDa) in AAV-treated and vehicle-treated muscles in CTRL and *Neb* cKO mice. Two AAV dosages were used. (1×, 1 × 10^11^ vg; 3×, 3 × 10^11^ vg). **c**, **d** FLAG expression at 1× (**c**) and 3× (**d**) dosages of the AAV in TC (top) and EDL (bottom). AAV treatment resulted in construct expression in both TC and EDL of CTRL and *Neb* cKO mice. (1×: *n* = 5, 6 mice; 3×: 4, 9 mice). **e** Analysis of AAV dosage effect (1× and 3×) and genotype (CTRL and *Neb* cKO) in TC (top) and EDL (bottom) muscles. A two-way ANOVA reveals no dosage effect but a genotype effect with higher expression in *Neb* cKO mice compared to CTRL mice. ns, non-significant (comparison between 1× and 3× dose). TC, tibialis cranialis; EDL, extensor digitorum longus; L, left (PBS-injected); R, right (AAV-injected)

A 3X-FLAG and HA tagged version of the murine nebulin Z-disk region consisting of super-repeat 25, repeat actin-binding modules 184-206, the serine-rich region, and the SH3 domain (see Fig. 1a) was codon optimized for murine expression and synthesized. This region consists of 1181 amino acids spanning from exons 125 to 157 of the murine nebulin sequence (NM_0.10889.1) and is 135 kDa without the tags. The human equivalent of this region is exons 146 to 183, 1334 residues estimated to be 153 kDa. All exons were expressed to mimic expression in slow-type muscles. The construct was subcloned into a pAAV cassette with AAV2 inverted terminal repeats, the CK8e muscle-specific promoter [50], a β-globin/IgG chimeric intron (Promega), and a synthetic polyadenylation sequence [51] engineered from the rabbit beta-globin gene. The pAAV and a packaging/helper plasmid pDGM6 were then co-transfected into HEK293 cells to make recombinant AAV6-CK8-mNebulin Z-disk as previously described [52]. Titers were determined by Southern blot and qPCR using primers and/or a probe that recognizes the CK8e promoter.

### Intramuscular injection of the AAV construct

Conditional nebulin knockout mice [36] along with littermate controls were injected with the Z-disk AAV construct at weaning age (~ 21 days postnatal). A dosage of either 1 × 10^11^ vg (1× dosage) or 3 × 10^11^ vg (3× dosage) was injected intramuscularly into the anterior compartment of the lower hindlimb. The tibialis cranialis (TC) muscle was targeted, but both the tibialis cranialis and the extensor digitorum longus (EDL) are bathed in the AAV during in this protocol [53]. For the sake of consistency, the left hindlimb was always treated with the vehicle (PBS), while the right hindlimb was always treated with the AAV. Mice were sacrificed 1 month later and both TC and EDL muscles were taken for studies. All animal procedures were approved by the University of Arizona Institutional Animal Care and Use Committee.

### Tissue collection

Mice were sacrificed according to approved protocols. Mice were weighed before being anesthetized via isofluorane. A toe pinch was used to verify complete anesthesia before cervical dislocation and diaphragm puncture. TC and EDL muscles were dissected, with both left and right EDL muscles being used for whole-muscle mechanical studies. TC muscles were split into pieces, with one part being flash frozen for protein studies and the other part being demembranated for immunofluorescence and electron microscopy studies. Following whole-muscle mechanical studies, EDL muscles were also frozen in liquid nitrogen. Frozen tissues were stored at − 80 °C. The lengths of left and right tibias were measured via electronic caliper before being averaged. The average was used to normalize muscle weights.

### Sample preparation, gel electrophoresis, and Western blotting

Muscle samples were prepared as previously described [54]. Rapidly frozen tissues were ground into powder at liquid nitrogen temperature via glass Dounce tissue homogenizers pre-chilled in liquid nitrogen. Tissue powder was allowed to equilibrate in a − 20 °C refrigerator for 20 min before 50% glycerol and a urea buffer were added in a 1:40:40, sample (mg):glycerol (μL):urea (μL), ratio. Glycerol solution was made using H_2_O, glycerol, and a mix of inhibitors ((in mM) 0.04 E− 64, 0.16 leupeptin, 0.5 PMSF). Urea buffer contained 8 M urea, 2 M thiourea, 50 mM tris-HCl, 75 mM dithiothreitol, 3% SDS w/v, and 0.03% bromophenol blue, with a pH of 6.8. The solution was mixed and incubated at 60 °C for 10 min before being aliquoted and flash frozen in liquid nitrogen.

Myosin heavy chain gels were performed on 8% acrylamide gels as previously described, run for 24 h at 275 V before being stained with Coomassie blue [55]. Gels for the protein pulldown used 8% SDS-PAGE followed by staining with Coomassie blue. Western blots for full-length and Z-disk fragment nebulin were run with 0.8% agarose gels run for 15 mA/gel for 2 h 35 min before being transferred to a PVDF membrane using a semi-dry transfer unit (Bio-Rad, Hercules, CA, USA). A multicolor broad range protein ladder (Thermo Fisher) was used to locate the Z-disk fragment in these blots. Western blots for KLHL41 and NRAP were run using 10% SDS-PAGE before being transferred to a PVDF membrane. All blots were initially stained with Ponceau S for protein visualization. Membranes were then blocked and incubated overnight at 4 °C with the appropriate primary antibodies. The nebulin SH3 antibody was provided by Dr. Siegfried Labeit (1:200, rabbit). Additionally, primary antibodies to KLHL41 (1:400 rabbit ab66605, Abcam) and NRAP (1:1000 rabbit ab122427, Abcam) were used. Western blots for full-length nebulin and Z-disk nebulin fragment were normalized with MHC visualized through Ponceau S. Blots run for KLHL41 and NRAP were normalized to GAPDH (1:2000 mouse #GA1R, Thermo Fisher). Secondary antibodies used were conjugated with infrared fluorophores for detection (1:20000 goat anti-rabbit CF680, Biotium, and 1:20000 goat anti-mouse CF790, Biotium). Infrared Western blot was analyzed using an Odyssey CLx Imaging System (Li-Cor Biosciences, NE, USA). MHC viewed through Ponceau S was quantified via One-D scan EX (Scanalytics Inc., Rockville, MD, USA).

### Sample preparation for immunofluorescence and electron microscopy

Fiber skinning was performed as previously described [31]. TC muscles were split and placed in relaxing solution (in mM: 40 BES, 10 EGTA, 6.56 MgCl_2_, 5.88 Na-ATP, 46.35 K-propionate, 15 creatine phosphate at pH 7.0) with 1% triton X-100 for demembranation (skinning). At all steps, protease inhibitors were added just prior to use. Muscles were placed on a 2D rocker overnight at 4 °C. Following skinning, muscles were washed with relaxing solution alone to remove excess triton X-100. Then, samples were placed in 50% glycerol/relaxing solution first overnight, then stored at − 20 °C. To obtain fiber bundles, skinned muscles were placed in sylgard dishes containing additional 50% glycerol/relaxing solution with protease inhibitors and then bundles were carefully dissected from the muscle. Bundles were held at both ends with aluminum T-clips and pinned at ~ 30% past slack length.

For immunofluorescence, bundles were fixed overnight at 4 °C in a 10% formalin (4% formaldehyde) solution. Following fixation, bundles were washed with PBS before being removed from the T-clips and embedded in OCT. Six-micrometer thick longitudinal sections were collected onto glass slides. These sections were fixed again in triton X-100 and blocked with normal donkey serum as described above. Primary antibodies to the HA tag (1:200 rabbit C29F4, Cell Signaling) and phalloidin 488 (1:2000 A12379, Invitrogen) were applied for an overnight incubation at 4 °C. Fluorescent secondary antibodies were applied after post-primary washes: polyclonal Alexa Fluor 594-conjugated goat anti-rabbit (1:600 IgG (H+L) A11012, Thermo Fisher) and phalloidin 488. Deconvolution microscopy was performed using a Deltavision RT deconvolution microscope (Applied Precision) with an inverted microscope (IX70, Olympus) and the softWoRx program.

For electron microscopy, fiber bundles were briefly fixed in a 3% paraformaldehyde solution (3% PF, 2% glutaraldehyde, 0.03% tannic acid in PBS (0.01 M, pH 7.2)) for 45 min at 4 °C. Then, fixative was washed off with PBS and replaced with a 1% w/v OsO_4_ solution in PBS. After this, fixed samples were gradually dehydrated in a series of ethanol washes, starting at 70% ethanol and ending with a mix of pure ethanol and propylene oxide. Then, samples were infiltrated with resin (araldite/embed813) and then finally embedded in BEEM capsules (Ted Pella) for sectioning. Sections were taken at 60 μm with a diamond knife set parallel to the fiber orientation. These sections were then incubated with 1% potassium permanganate followed by 0.25% lead citrate for contrast. Images were taken with transmission electron microscopy (FEI/Phillips CM12). Sarcomere density profiles were obtained via FIJI (ImageJ) and plot profiles were processed using the Fityk software.

### Intact muscle mechanics

Whole-muscle mechanics were done using an Aurora Scientific 1200A isolated muscle system [56, 57]. Briefly, both left and right EDL muscles were carefully extracted, keeping proximal and distal tendons intact. Silk suture loops (4–0 diameter) were tied to each tendon and the muscle was attached to both a servomotor-force transducer and a stationary hook. Muscles were submerged in an oxygenated Krebs-Ringer bicarbonate solution at 30 °C (in mM: 137 NaCl, 5 KCl, 1 NaH_2_PO_4_·H_2_O, 24 NaHCO_3_, 2 CaCl_2_·2H_2_O, 1 MgSO_4_·7H_2_O, 11 glucose, pH 7.5). Optimal length (*L*_0_) was found by first performing a tetanus to remove any slack in the sutures, allowing the muscle to recover, and then increasing length until twitch forces plateaued. Force-frequency relationship was determined by subjecting muscles to increasing stimulation frequencies (in Hz: 1, 10, 20, 40, 60, 80, 100, 150 for soleus with an additional 200 for EDL). Muscles were allowed to recover for 30, 30, 60, 90, 120, 120, 120, 120 s between subsequent stimulations. Fatigue protocol was performed as follows: 1 s submaximal stimulation at 60 Hz, followed by 2 s of rest, repeated 75 times. No protocols were performed following the fatigue protocol. Force obtained (converted to mN) was normalized to the physiological cross-sectional area (PCSA) through the following equation: PCSA = mass (mg)/[muscle density (mg/mm^3^) × fiber length (mm)]. The physiological density of muscle is 1.056 and fiber length was found utilizing a fiber length to muscle length ratio, 0.72 for soleus and 0.51 for EDL [58].

### Statistics

One-variable comparisons were Student’s paired *t* tests performed between vehicle-treated and AAV-treated muscles. Bar graphs are formatted as mean ± SEM. Where applicable, two-way ANOVA or repeated-measure two-way ANOVA was performed to include variables such as treatment, muscle type, or genotype. For the force-frequency relationship, the Hill equation was used to fit the sigmoidal curve. For both asterisks (*) and hashtags (#), significance is as follows: *(#)*p* < 0.05, **(##)*p* < 0.01, ***(###)*p* < 0.001, ****(####)*p* < 0.0001. Statistical analysis was performed using GraphPad Prism 7.04 software (GraphPad Software Inc., La Jolla, CA, USA).

## Results

### Expression of a nebulin Z-disk fragment in control (CTRL) and nebulin-deficient (*Neb* cKO) mice

To test the effect of expressing the Z-disk region of nebulin on the structure and function of the skeletal muscle sarcomere, an adeno-associated viral (AAV) vector expressing the Z-disk region plus nebulin’s final super-repeat was created (Fig. 1a). This AAV construct, estimated to be 139.9 kDa, was injected into the anterior compartment of the lower hindlimb of the mouse as previously described [31]. This allows for the AAV to enter muscle fibers and the construct to be expressed in both the tibialis cranialis (TC) and the extensor digitorum longus (EDL) muscles [59]. The Z-disk region was introduced into a conditional nebulin knockout mouse model (*Neb* cKO) at weaning age (~ 21 days post-natal). This model removes the floxed start codon of nebulin using a *Cre*-recombinase, which is expressed through the activation of the muscle creatine kinase promoter (MCK-Cre). This removal results in ~ 50% nebulin (relative to controls) at weaning age which decreases to < 5% 2 weeks later [36]. Injecting this AAV construct at weaning age allows the diminishing full-length nebulin protein to be replaced by the Z-disk fragment before severe pathology ensues. Mice that do not express MCK-Cre (e.g., containing a floxed nebulin gene) were also injected with the construct and functioned as negative controls (CTRL). These negative control mice have previously been shown to be identical to wildtype mice [32, 36]. In both genotypes, the right hindlimb was always injected with the AAV and the left leg with the PBS (AAV vehicle). Using two AAV dosages (detailed in the “Materials and methods” section), the efficacy of this construct was also evaluated. Nine CTRL mice and 12 *Neb* cKO mice were used for the 1× dosage group, while 4 CTRL mice and 9 *Neb* cKO mice were used for the 3× dosage group. Following a 1-month incubation time, mice were sacrificed. Left (L) and right (R) TC and EDL muscles were dissected and used for experiments.

Z-disk fragment expression was determined via a FLAG tag primary antibody in Western blot studies (Fig. 1b). This revealed strong signals in the AAV-treated muscles (RTC and REDL) and no signal in the vehicle-treated muscles (LTC and LEDL). These results were similar in both AAV dosage groups. Expression levels were quantified relative to GAPDH, revealing significant AAV-induced increases in FLAG tag levels. This was observed in both TC and EDL muscles from CTRL and *Neb* cKO mice at the 1× (Fig. 1c) and 3× (Fig. 1d) AAV dose. These data were also analyzed with a two-way ANOVA, comparing AAV dose (1× vs 3×) and genotype (CTRL and *Neb* cKO). This showed that while the dose did not affect construct expression, genotype did. In both AAV-treated TCs (Fig. 1e, top) and EDLs (Fig. 1e, bottom), *Neb* cKO muscles expressed significantly more Z-disk fragment than the CTRL muscles. The lack of a dosage effect implies that muscles of both genotypes contain a maximal amount of nebulin fragment following the lower dose and that higher dosages do not result in increased protein levels. The increased Z-disk fragment levels in *Neb* cKO muscles relative to CTRL muscles suggest that they express and/or stabilize the fragment better than CTRL muscles.

Muscle weights from each of these treatment groups were also analyzed (Additional file 1: Figure S1A–B). With the exception of the *Neb* cKO TCs in the 1× dosage group where a small weight reduction occurred (Additional file 1: Figure S1A, red), muscle weights were not significantly affected by the AAV treatment. This data suggests that inducing expression of a construct containing the Z-disk region of nebulin does not have a noticeable therapeutic effect on muscle weights. Further supporting this finding, the physiological cross-sectional areas (PCSAs) of the EDL muscles used in the functional studies (see below) was also unchanged (Additional file 1: Figure S1C).

In summary, we successfully expressed the Z-disk region of nebulin in both TC and EDL muscles of CTRL and *Neb* cKO mice. AAV-treated muscles exhibit a significant Z-disk fragment expression, but this expression was not changed when the dosage was increased threefold, implying the 1× dosage group was sufficient. *Neb* cKO muscles consistently expressed higher Z-disk fragment levels, suggesting that the Z-disk fragment may integrate into the nebulin-deficient sarcomere more readily. Because there is no definitive change in muscle weight or PCSA, effects of this AAV may be more on a molecular or functional level. As such, localization and functional studies were performed next.

### Quantification of Z-disk nebulin fragment and full-length nebulin in CTRL and *Neb* cKO mice

**Fig. 2:**
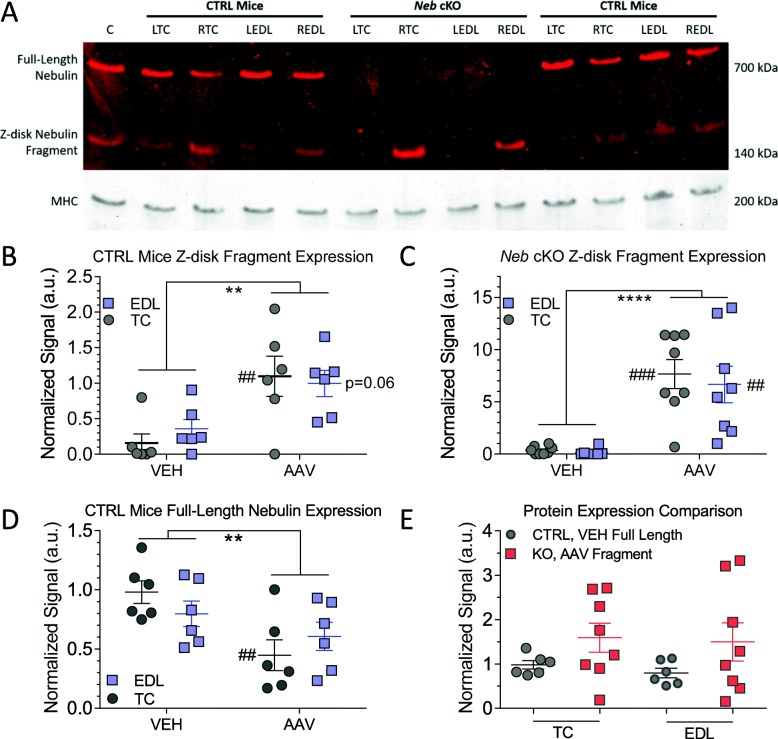
Expression of full-length nebulin and Z-disk fragment in AAV-treated muscles. **a** Example Western blot using SH3 antibody that labels both full-length and the Z-disk AAV nebulin. Full-length nebulin is only detected in the CTRL mice, in both the vehicle-treated (LTC and LEDL) and AAV-treated muscles (RTC and REDL). The Z-disk fragment is detected in AAV-treated muscles (RTC and REDL) of both genotypes. (Lane marked with ‘C’: AAV-treated muscle that was loaded on all gels and that functioned as a universal control sample allowing for comparison of multiple blots.). **b**, **c** Z-disk fragment expression in vehicle-treated and AAV-treated muscles in CTRL (**b**) and *Neb* cKO (**c**) mice. Repeated-measure 2-way ANOVA shows that Z-disk fragment is significantly increased in AAV-treated CTRL and *Neb* cKO muscles. Significance between vehicle-and AAV-treated muscles marked with #. **d** Full-length nebulin expression in vehicle-treated and AAV-treated muscles in CTRL mice. Repeated-measure 2-way ANOVA shows that the treatment has a significant effect (*) and that AAV-treated TC muscles experience a significant loss of full-length nebulin (#). **e** Comparison of Z-disk fragment expression in AAV-treated *Neb* cKO mice to full-length nebulin in vehicle-treated CTRL mice. Treatment results in a Z-disk fragment expression that is comparable to full-length nebulin in CTRL mice, in both TC and EDL. (Muscles treated with the 1× AAV dose). (*n* = 6, 8 mice)

To simultaneously determine the expression of the Z-disk nebulin fragment and full-length nebulin, an antibody to nebulin’s SH3 domain, present in both proteins of interest, was used (Fig. 1a). The SH3 antibody strongly labels the Z-disk fragment in AAV-treated muscles (RTC, REDL) with relatively weak labeling in AAV-treated CTRL mice and stronger labeling in AAV-treated *Neb* cKO mice (Fig. 2a, Z-disk nebulin fragment). As expected, this antibody also strongly labels full-length nebulin in CTRL mice with no detectable signal present in the *Neb* cKO mice (Fig. 2a, full-length nebulin).

Because no significant difference in protein expression was detected between the two dosage groups (Fig. 1e), the following analyses were only performed on tissues treated with the 1× dosage of the AAV construct. The Z-disk nebulin fragment signal was quantified and normalized to myosin heavy chain (MHC). We expected the Z-disk nebulin fragment signal to resemble FLAG tag signal (Fig. 1b). But due to the resolution of the gels used, a background band in PBS-injected muscle overlapped with the Z-disk nebulin fragment signal. This background was subtracted in the following analyses using the average of signal from the fragment-negative lanes. When individual muscle types in CTRL mice were analyzed with a repeated-measure two-way ANOVA, a significant increase was found in the TC muscles with a trending increase in the EDL muscles (Fig. 2b, hashtags). Similarly, *Neb* cKO muscles saw a significant increase in both muscle types (Fig. 2c, hashtags). Through this analysis, AAV treatment was also shown to have a significant effect on Z-disk fragment expression in both genotypes, regardless of muscle type (Fig. 2b and c, asterisks). These data indicate that the Z-disk fragment is highly expressed in treated muscle, with *Neb* cKO mice exhibiting a stronger signal.

Considering the significant expression of this Z-disk fragment in AAV-treated CTRL mice, it was necessary to study its effect on full-length nebulin expression. Because *Neb* cKO mice do not have detectable full-length nebulin in either vehicle-treated or AAV-treated muscles, this analysis was only performed on CTRL mice. In this quantification, repeated-measure two-way ANOVA showed that AAV treatment resulted in a significant decrease of full-length nebulin expression (Fig. 2d, asterisks). Accounting for individual muscle types, it was found that the TC muscles specifically experience a significant decrease in full-length nebulin expression (Fig. 2d, hashtags). This data shows that injecting healthy tissues with the Z-disk fragment AAV displaces full-length nebulin over time, resulting in less full-length nebulin protein.

Finally, the expression of Z-disk fragment in AAV-treated *Neb* cKO mice was compared to that of full-length nebulin in vehicle-treated CTRL mice. This analysis revealed that expression of the Z-disk fragment in both TC and EDL muscles of AAV-treated *Neb* cKO mice was similar to that of full-length nebulin in vehicle-treated CTRL mice (Fig. 2e). This finding indicates that, by treating *Neb* cKO muscles with the Z-disk fragment AAV, nebulin-deficient muscles can produce the Z-disk fragment at levels comparable to full-length nebulin in vehicle-treated CTRL muscles.

### Localization of the Z-disk nebulin fragment and analysis of sarcomere structure

**Fig. 3:**
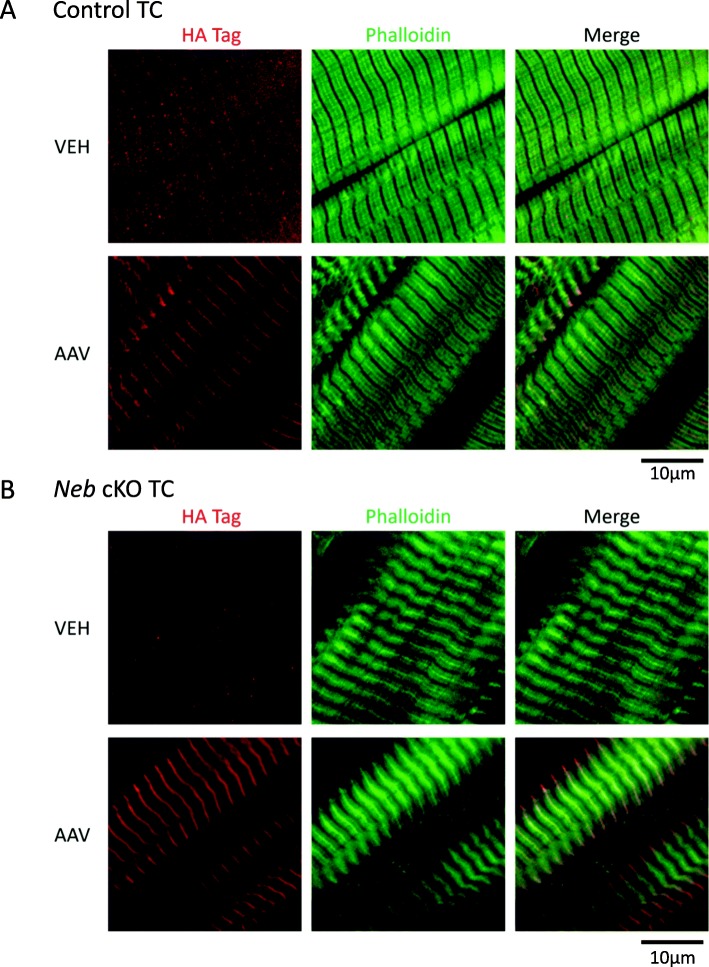
Localization of the Z-disk AAV construct. Z-disk AAV construct localization in skinned TC fibers from CTRL (**a**) and *Neb* cKO (**b**) mice. In both genotypes, Z-disk labeling is seen in the AAV-treated muscle. (Shown results were obtained with 1× AAV dosage. Identical results were obtained in the 3× dosage muscles.) (*n* = 3 mice)

To determine if the Z-disk nebulin fragment targeted the expected location in the sarcomere, immunofluorescence was performed on TC muscles from the 1× dosage group using an HA-tag antibody. Regardless of genotype, the Z-disk fragment is found to localize exclusively to the Z-disks in AAV-treated TC muscles (Fig. 3a, b—AAV). Vehicle-treated TC muscles showed no HA-tag signal (Fig. 3a, b—VEH). Identical results were found in the 3× dosage group (Additional file 1: Figure S2A, B). These data show that the AAV construct utilized in this study can successfully incorporate into the Z-disks at both dosages.

**Fig. 4:**
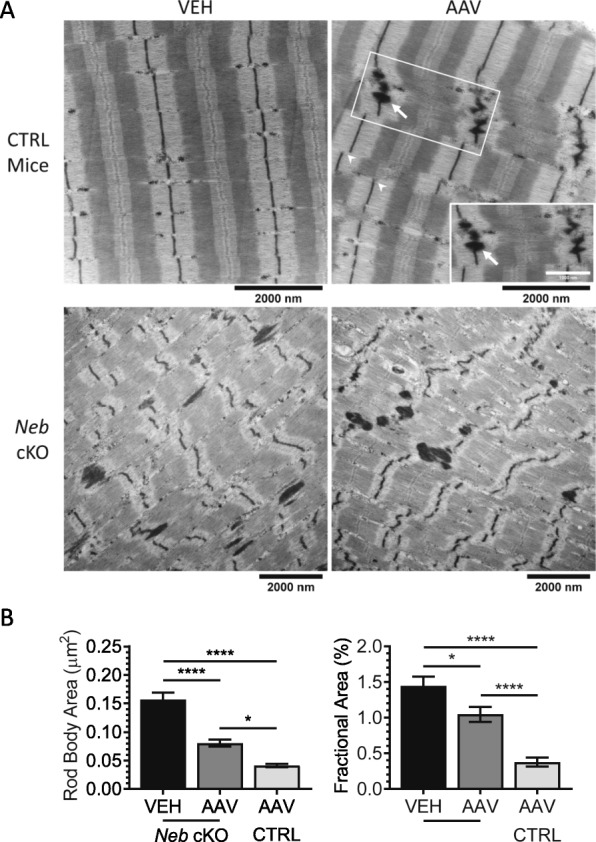
Ultrastructural analysis. **a** Representative images from skinned TC muscles for each treatment group. Z-disk misalignment (arrowheads) and rod bodies (arrows, insert) identified in AAV-treated CTRL mice. Black scale bar applicable to all images aside from the insert (white scale bar: 1000 nm). **b** Characterization of observed nemaline rod bodies. Left: rod body size, right: fractional area in muscle occupied by rod bodies. AAV treatment of *Neb* cKO mice reduces rod body size. Treatment in CTRL mice results in the formation of new rod bodies. (*n* = 103–191 rod bodies)

To study the effects of the AAV on sarcomere structure, a transmission electron microscopy (TEM) study was also performed. Notably, AAV-treated CTRL TC muscles had misaligned myofibrils (Fig. 4a, CTRL, arrowheads) and sporadic broadening of the Z-disks that resembled developing nemaline rod bodies (Fig. 4a, CTRL, arrows). No obvious structural changes were found in the *Neb* cKO TC muscles, with overall structure remaining disorganized and nemaline rod bodies frequently observed (Fig. 4a, *Neb* cKO). However, when the observed rod bodies were characterized, AAV-treated *Neb* cKO TC muscles were found to have significantly smaller rod bodies, with AAV-treated CTRL TC muscles being even smaller (Fig. 4b, left). Similarly, when the fractional area occupied by rod bodies was quantified, we see a decrease correlating with the decreased rod body size (Fig. 4b, right). This suggests that AAV treatment is affecting the formation of rod bodies, specifically in *Neb* cKO muscles.

**Fig. 5:**
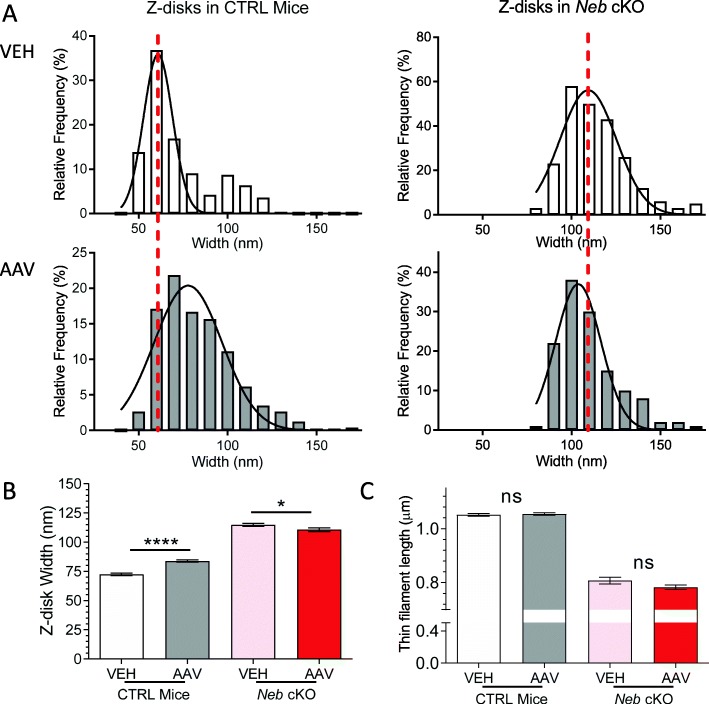
Z-disk width and thin filament length analysis. **a** Z-disk width distribution in CTRL (left) and *Neb* cKO (right) TC muscle. In CTRL mice, the Gaussian curve fit is significantly right-shifted in AAV-treated muscles by ~ 17 nm (*p* < 0.0001). (Note that while vehicle-treated CTRL mice have a double peak, it was not sufficient to qualify this data for a sum of two Gaussians curve fits.) In *Neb* cKO mice, the Gaussian curve fit is significantly left-shifted by ~ 6 nm (p < 0.0001). Measurements were made using electron microscopy images. (Analysis restricted to sarcomeres devoid of rod bodies. 8–17 fibers from 2 CTRL and 2 *Neb* cKO mice, with an equal number of Z-disks sampled from each fiber.) **b** Average Z-disk widths. Treatment with the Z-disk AAV causes a significant widening of the Z-disks in CTRL mice and a slight, but significant, decrease in the *Neb* cKO mice. **c** Thin filament lengths. No effect of AAV treatment on thin filament length in either genotype. (Measurements based on phalloidin-labeled sections imaged by deconvolution microscopy. In these experiments, a 1× AAV dosage was used. SL: 2.57 ± 0.05 μm (*n* = 45; CTRL, VEH), 2.57 ± 0.04 μm (*n* = 33; CTRL, AAV); 2.18 ± 0.16 μm (*n* = 41; *Neb* cKO, VEH); 2.18 ± 0.13 μm (*n* = 79; *Neb* cKO, AAV). (Unpaired *t* tests with Welch’s correction were performed)

Because of the Z-disk localization exhibited by the Z-disk fragment (above), and the previous finding that deleting a small portion of nebulin’s Z-disk region alters Z-disk widths [31], the widths of the Z-disks in these samples were also analyzed (sarcomeres with nemaline rod bodies were avoided). While differences were difficult to discern even at high magnification (Additional file 1: Figure S3), an even sampling across multiple unique fibers of two mice within each treatment group revealed the changes in widths. AAV-treated CTRL TC muscles had significantly wider Z-disks (Fig. 5a, left; b). However, AAV-treated *Neb* cKO muscles experienced a reduction in Z-disk widths (Fig. 5a, right; b). As widening Z-disks is known to be a marker of pathology [32], it appears that the AAV-treated CTRL mice transition toward a myopathic phenotype, while the disease progression of *Neb* cKO mice is being slowed.

It is well-known that nebulin plays a role in thin filament length regulation [32, 33, 36, 60]. Thus, the effect of AAV treatment on thin filament length was studied using phalloidin-labeled sections of TC muscles and deconvolution microscopy. Thin filaments were shorter in *Neb* cKO mice (Fig. 5c), confirming previous studies [32, 33, 36]. However, no thin filament length differences were found between vehicle-treated and AAV-treated muscles (Fig. 5c). These data supports the previous finding that nebulin’s Z-disk region primarily regulates Z-disk widths and has no effect on thin filament lengths [31].

### Myosin heavy chain expression in response to treatment with the Z-disk fragment AAV

**Fig. 6:**
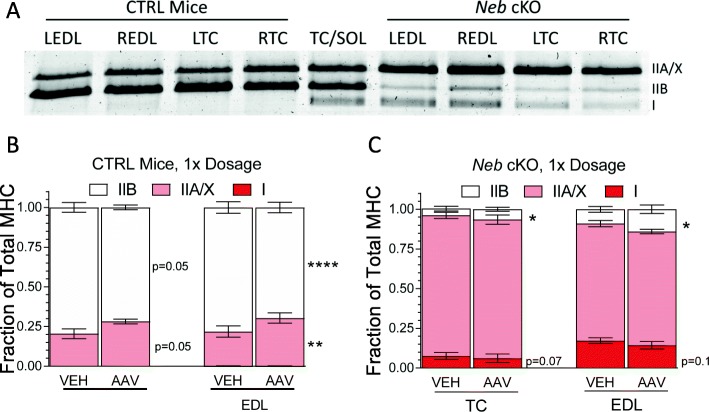
Myosin heavy chain composition. **a** Representative acrylamide gel depicting separation of myosin heavy chain (MHC) isoforms. Left four columns are vehicle-treated (LEDL, LTC) and AAV-treated (REDL, RTC) CTRL mice. Middle column is a mixture of TC and soleus muscle from a C57BL/6 mouse serving as a marker for the isoforms. Right four columns are vehicle-treated (LEDL, LTC) and AAV-treated (REDL, RTC) *Neb* cKO mice. **b** Quantification of MHC composition in vehicle-treated and AAV-treated CTRL TCs and EDLs (1× dosage). A significant increase in Type IIA/X MHC and a significant decrease in Type IIB MHC occur in the EDLs. TCs trend in the same direction (*n* = 6 mice). **c** Quantification of MHC composition in vehicle-treated and AAV-treated *Neb* cKO TCs and EDLs (1× dosage). A significant increase in Type IIB MHC and trend toward a reduced MHC I (*n* = 9 mice). (Paired *t* tests were used in these analyses)

Nemaline myopathy is also associated with a shift toward slower fiber types [36, 61–63]. AAV-treated muscles (REDL and RTC) and vehicle-treated muscles (LEDL and LTC) of both CTRL and *Neb* cKO in the 1× AAV dosage group were run on acrylamide gels in order to visualize the different MHC isoforms present in skeletal muscle (Fig. 6a). Consistent with previous work [36], *Neb* cKO mice express slower myosins (less IIB, more I and IIA/X). Quantification of the MHC composition in AAV-treated CTRL mice showed that in both TC and EDL muscles, composition had shifted toward slower populations compared to vehicle-treated muscles. Through paired *t* tests, EDL muscles showed a significant reduction of type IIB MHC and a significant increase in type IIA/X MHC, with the same changes trending in the TC muscles (Fig. 6b). These changes support the above findings that CTRL muscles are becoming myopathic. Conversely, AAV-treated *Neb* cKO mice shifted toward a faster composition. In both TC and EDL muscles, paired *t* tests found a significant increase in type IIB MHC and trending decreases in type I MHC (Fig. 6c). These results were replicated in the 3× dosage group (Additional file 1: Figure S4). Thus, treatment of nebulin-deficient muscles with the Z-disk fragment AAV slows disease progression.

### Effects of the Z-disk fragment expression on isometric force

**Fig. 7:**
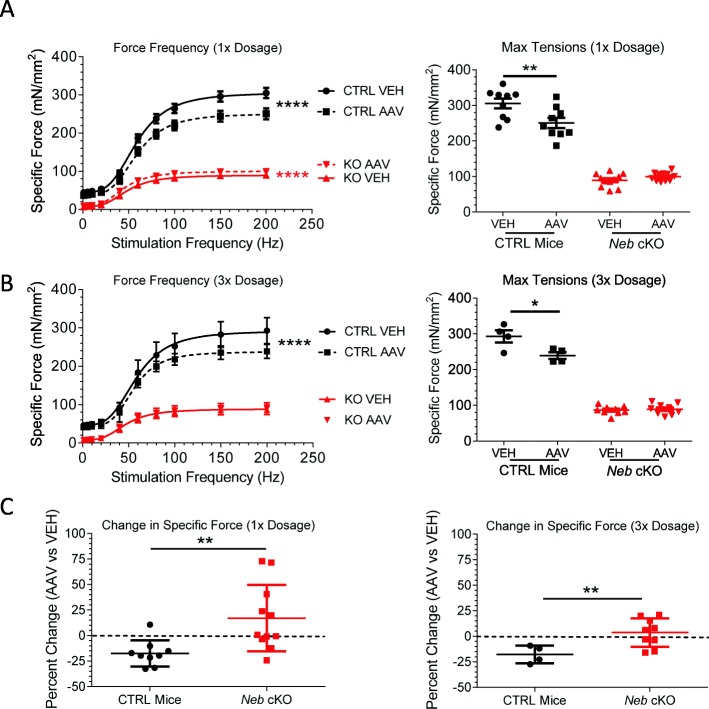
Specific force in AAV-treated and vehicle-treated EDLs of CTRL and *Neb* cKO mice. **a**, **b** Force-frequency curves for Z-disk AAV mice at the 1× (**a**) and 3× (**b**) dosage. Left: There was a significant decrease in the force-frequency curve fit for the CTRL mice at both doses and a slight, but significant, increase for the *Neb* cKO mice at the 1× dose. That increase in the *Neb* cKO curve fit was lost in the 3× dose. Right: Quantitation of maximal (200 Hz) tetanic force. Paired *t* tests at both doses show a significant decrease in CTRL mice, but no significant increase in *Neb* cKO mice. (Paired *t* tests were used to compare maximal tetanic force.) **c** Percent change between AAV-treated and vehicle-treated EDL in the 1× (left) and 3× (right) dosage groups. There is a significant difference between the response of CTRL mice and the response of *Neb* cKO mice to the treatment in both dosage groups. Analyzing the change in a one-sample *t* test with a reference value of 0% change shows that AAV treatment does not affect force production in *Neb* cKO mice (*p* = 0.11 (1× dose) and 0.44 (3× dose)). (1×: *n* = 9, 11 mice; 3×: *n* = 4, 9 mice)

To characterize the effects of Z-disk nebulin fragment expression on whole-muscle function, in vitro muscle mechanical studies were performed on both vehicle-treated and AAV-treated EDL muscles. Multiple stimulation frequencies were used to determine the isometric force-frequency relation. This relation was fit with a sigmoidal curve according to the Hill equation, and the differences in fit were compared. A significant force reduction was found when comparing the force-frequency curves of the CTRL muscles, both when using the 1× AAV dose (Fig. 7a, left, black symbols) and the 3× AAV dose (Fig. 7b, left, black symbols). When force production was compared at individual frequencies using a repeated-measure two-way ANOVA, forces were significantly less starting at 60 Hz in the 1× dosage group (Additional file 1: Table S1A) and significantly less starting at 150 Hz in the 3× dosage group (Additional file 1: Table 1B). The maximal tetanic force exhibited on average a 17.4% loss in the 1× dosage group and a 18.4% loss in the 3× dosage group (Fig. 7a and b, right, black symbols).

AAV-treated *Neb* cKO mice were found to have a slightly higher sigmoidal curve fit in the 1× dosage group (Fig. 7a, left, red symbols), but the fits were not different in the 3× dosage group (Fig. 7b, left, red symbols). When force production was compared at individual frequencies using a repeated-measure two-way ANOVA, none of the comparisons were found to be significantly different (Additional file 1: Table S1). Maximal tetanic forces were also not different (Fig. 7a and b, right, red symbols). In comparing the effects of the AAV on maximal tetanic force of the two genotypes, a significant difference was found (Fig. 7c, asterisks). Additionally, the change in maximal tensions was analyzed using a one-sample *t* test to determine whether the mean results differ from zero. In the 1× dosage group, CTRL mice were found to differ significantly from zero (*p* = 0.004) while *Neb* cKO mice were not significantly different (*p* = 0.11). The same results were found in the 3× dosage group, with CTRL mice differing significantly (*p* = 0.026) and *Neb* cKO muscles not differing significantly (*p* = 0.44). Though *Neb* cKO mice do not exhibit the distinct negative effect that is present in AAV-treated CTRL mice, neither is there a significant positive effect on maximal tension.

We also evaluated the sensitivity to increasing frequencies by normalizing the force-frequency sigmoidal curves to the maximal force within each experiment (Additional file 1: Figure S5A). The normalized sigmoidal curves were left-shifted in the *Neb* cKO mice compared to the CTRL mice. This might reflect the increased number of Type I and IIA fibers in the EDL muscle from *Neb* cKO mice compared to CTRL mice [36]. There is also a further leftward shift in AAV-treated *Neb* cKO EDL muscles in the 1× dosage group, but this change is not observed in the 3× dosage group (Additional file 1: Figure S5).

### Changes in force kinetics and fatigue

We also evaluated the contraction kinetics and fatigability of the EDL muscle. The time to maximal force in both a twitch and maximal tetanus (200 Hz) were analyzed (Additional file 1: Figure S6A). While AAV-treated *Neb* cKO muscles of the 3× dosage group had a significantly longer twitch time, no differences were observed in the 1× dosage group (Additional file 1: Figure S6A, left). On the other hand, AAV-treated *Neb* cKO muscles of the 1× dosage group took longer to reach maximal force during a tetanus but showed no change in the 3× dosage group (Additional file 1: Figure S6A, right). When analyzing the relaxation times of both the twitch and the maximal tetanus, minimal changes were observed in the twitch of *Neb* cKO muscles in the 3× dosage group (Additional file 1: Figure S6B, left). However, relaxation times following a tetanus were significantly increased (a trending increase was found in the case of *Neb* cKO muscles in the 3× dosage group) (Additional file 1: Figure S6B, right). When subjecting this data to a two-way ANOVA, AAV treatment has a significant effect on the relaxation time (1× dosage: *p* = 0.0004, 3× dosage: *p* = 0.0044). Based on this kinetics data, it can be concluded that expressing the Z-disk region of nebulin in muscles has the greatest effect on tetani, where it slows relaxation.

**Fig. 8:**
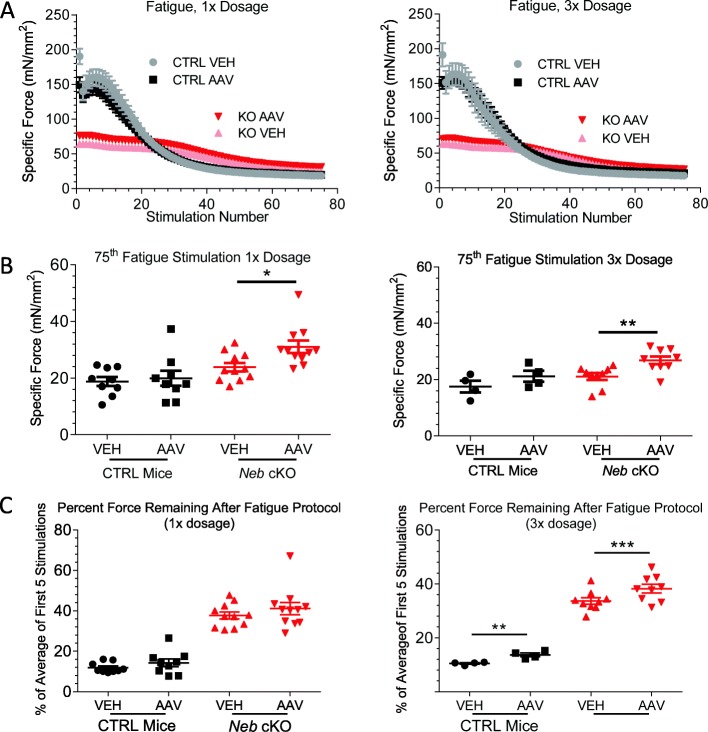
Fatigability of muscles treated with a 1× and 3× dose of nebulin’s Z-disk region. **a** Fatigue protocol consisting of 75 stimulations (1-s 60 Hz stimulation, 2-s rest) performed in 1× (left) and 3× (right) dosage groups. Notably, *Neb* cKO mice are more fatigue resistant due to previously reported changes in fiber-type composition [36]. **b** Quantification of force produced at the final stimulation of the 1× (left) and 3× (right) dosage group fatigue protocols. In both dosage groups, *Neb* cKO mice are found to produce more force at the end of the protocol (paired *t* test). **c** Force produced during the final fatigue stimulation as a percentage of the average of the force produced in the first five stimulations in 1× (left) and 3× (right) dosage groups. Higher percentage indicates resistance to fatigue. (1×: *n* = 9, 11 mice; 3×: *n* = 4, 9 mice)

By subjecting muscles to repeated submaximal tetanic stimulations with minimal time for rest (see the “Materials and methods” section), muscle fatigue was quantified. The fatigue response of CTRL EDL muscles was not significantly altered when the Z-disk fragment was introduced to the muscles (Fig. 8a, gray and black symbols). Comparisons performed at each individual stimulation found no significant difference between the forces produced by vehicle-treated and AAV-treated CTRL EDL muscles. On the other hand, *Neb* cKO mice produce less force but experience an increased resistance to fatigue-induced force loss (Fig. 8a, pink symbols). Like with the CTRL mice, AAV treatment does not alter the fatigue response (Fig. 8a, red symbols). However, when the force produced at the 75th stimulations was compared (this is the end of the protocol), AAV-treated *Neb* cKO muscles produce more force (Fig. 8b, red symbols). The percent force remaining was also compared (Fig. 8c). In this analysis, only the muscles in the 3× dosage group had a significant increase in percent force remaining (Fig. 8c, right). The 1× dosage group showed no significant differences. This suggests that the higher dose of the AAV treatment improves fatigue resistance.

### Alterations in nebulin chaperone protein KLHL41 and downstream protease target NRAP

**Fig. 9:**
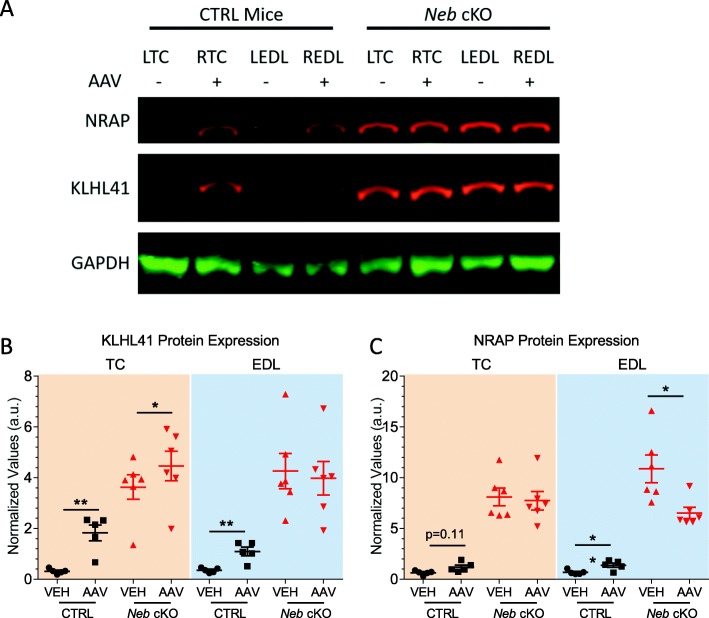
Expression of regulatory proteins in response to AAV treatment. **a** A representative Western blot depicting NRAP expression, KLHL41 expression, and GAPDH expression in vehicle-treated (LEDL, LTC) and AAV-treated (REDL, RTC) CTRL and *Neb* cKO mice. **b** Changes in KLHL41 expression following AAV treatment. Both CTRL and *Neb* cKO TC muscles experience an increase in protein expression. Similarly, CTRL EDL muscles experience an increase, but *Neb* cKO EDL muscles remain unchanged. **c** Changes in NRAP expression following AAV treatment. While CTRL TC muscles experience a trending increase toward higher expression, control EDL muscles have a significant increase in protein expression. *Neb* cKO EDL muscles experience a significant decrease in NRAP protein. (*n* = 5, 6 mice)

KLHL41 was previously determined to act as a chaperone and stabilizer for nebulin [64] and we investigated the effect of the Z-disk fragment on KLHL41 expression. In paired *t* tests, AAV-treated CTRL TC and EDL muscles experienced a significant increase in KLHL41 expression (Fig. 9a, b). While *Neb* cKO mice already have an increased baseline expression of KLHL41, it was further increased in AAV-treated TC muscles. The same changes in KLHL41 expression were found in the 3× dosage group (Additional file 1: Figure S7A). It was also recently reported that nebulin-related anchoring protein, NRAP, is associated with sarcomeric dysregulation and is regulated by KLHL41 [65]. Following Z-disk AAV treatment, CTRL TC muscles trended toward higher expression while CTRL EDL muscles experienced a significant increase in NRAP expression (Fig. 9c). There was no significant change in NRAP expression in of AAV-treated *Neb* cKO TC muscles, but there was a significant *decrease* in the AAV-treated EDL muscles. The same results were found in the 3× dosage group with the exception of the decrease in AAV-treated *Neb* cKO EDL muscles (Additional file 1: Figure S7B). These findings support that KLHL41 and NRAP contribute to the development of nemaline myopathy. However, treatment with the Z-disk fragment does not consistently alter their expression toward control levels.

### Z-disk fragment AAV treatment results in remodeling of the Z-disks with possible improvements in *Neb* cKO mice

From the changes observed in fiber-type composition and Z-disk structure, additional Z-disk proteins were quantified via Western blot (Additional file 1: Figure S8, S9). These included proteins involved in Z-disk stability (CapZ, BAG3, α-actinin, myotilin, Cypher/ZASP) and muscle differentiation/hypertrophy (myopalladin, CSRP3/muscle LIM protein, myozenin-1, myozenin-2). In every Z-disk protein analyzed, treatment with the Z-disk AAV had either a significant (*p* < 0.05) or trending effect toward higher protein expression in the CTRL muscles (Additional file 1: Figure S9). This strongly supports the Z-disk remodeling observed in the CTRL muscles, pointing to the involvement of nebulin’s Z-disk in sarcomeric regulation. And while the treatment response in *Neb* cKO mice was much more varied, there are a few points that stand out. Four proteins (CSPR3, myozenin-1, myozenin-2, and cypher-long) are either significantly decreased or trend toward down-regulation (Additional file 1: Figure S9B, G–I). When considering with the increases in the CTRL muscles, these results suggest that these four proteins may be reflective of disease pathology.

## Discussion

Treating nebulin-deficient muscles through therapeutic intervention has long been hindered by an incomplete understanding of nebulin’s function [39, 66, 67]. Moreover, recent studies have only expanded upon the complexities of nebulin function and its role in nemaline myopathy [4, 7, 31, 34–36, 40, 41, 67–71]. Specifically, we recently reported on the importance of nebulin’s C-terminal region in sarcomere stability [31]. In turn, recent attempts at therapies have focused primarily on improving contractility through pharmacological means [42–46]. Very few studies have attempted to restore nebulin expression due to its size, though one cell culture study in chick myocytes reported that introducing a shortened nebulin construct (termed ‘mini-nebulin’) into nebulin-deficient sarcomeres can be beneficial [72]. In this study, we critically extended this work through expressing the Z-disk region of nebulin in a mouse model of nebulin deficiency.

This study utilized the conditional nebulin knockout model (*Neb* cKO) [36] to circumvent early postnatal death caused by an embryonic nebulin knockout [32, 33]. In the present study, we created an AAV construct consisting of nebulin’s Z-disk region plus its final super-repeat (Fig. 1a). The goal was to have the Z-disk fragment replace the endogenous, full-length nebulin as full-length nebulin translation was halted in the *Neb* cKO mouse. This C-terminal region in human nebulin is known to have multiple alternative splicing isoforms [29] and contains many patient mutations [4]. However, mouse nebulin lacks the exonic equivalent of human exons 169–172 and exon 174, all of which are within this region of high alternative splicing [4, 29]. And an RNA-seq study into mouse nebulin expression recently revealed that slower fiber types, such as that present in the soleus, consistently express each of these exons (paper in preparation). Thus, the expression of all murine exons in this Z-disk fragment closely mimics nebulin isoforms in slow fiber types, the same fiber type that gets upregulated in nebulin knockout mice.

Our results showed that the Z-disk fragment successfully integrated into the Z-disks of the sarcomeres and that it was expressed in *Neb* cKO mice at levels comparable to full-length nebulin in vehicle-treated control muscles (Figs. 2e and 3). Additionally, the development of nemaline myopathy was slowed down as revealed by narrower Z-disk widths (Fig. 5a, right; b) and increased MHC IIB levels (Fig. 6c). These experiments emphasize that the C-terminal nebulin fragment encoded by this AAV has a strong affinity for the Z-disks of the sarcomeres and can displace full-length nebulin in control muscle. The details of these findings and their impact on treating nemaline myopathy are discussed below.

### Expression of the Z-disk fragment in CTRL and *Neb* cKO mice

AAV-treated muscles of both CTRL and *Neb* cKO mice highly expressed the Z-disk nebulin fragment (Fig. 1b), with the highest level in *Neb* cKO muscles (Fig. 1d, e). However, dosage was not found to have a significant effect on the expression of the construct (Fig. 1f). Specific quantification of Z-disk fragment expression using an antibody to nebulin’s SH3 domain revealed that not only do *Neb* cKO muscles achieve higher levels of this Z-disk fragment, but also the amount of Z-disk fragment expressed is comparable to that of full-length nebulin in vehicle-treated CTRL mice (Fig. 2). From this, we conclude that the Z-disk fragment is readily retained in nebulin-deficient *Neb* cKO muscles. With ~ 50% full-length nebulin at the time of AAV injection followed by a rapid reduction to near zero [36] the Z-disk fragment has little to compete with, allowing it to integrate efficiently into the sarcomeres. This finding also supports a recent study indicating that nebulin’s most C-terminal super-repeat has a strong affinity to actin filaments [73].

When the nebulin Z-disk fragment is expressed in CTRL tissues, the Z-disk fragment is found to compete with full-length nebulin. This resulted in both a significant decrease in full-length nebulin expression (Fig. 2d) and an increase in Z-disk fragment expression (Fig. 2b). This also resulted in a significant decrease in force production, discussed below. Furthermore, when mice were treated with an even lower dose of the Z-disk AAV (3.33 × 10^10^ vg), the force decrease remained the same. This implies that the AAV fragment has a stronger affinity for the Z-disks than native nebulin. Nebulin has a strict stoichiometry within skeletal muscle [74, 75] and the displaced full-length nebulin is likely degraded within CTRL muscles. With the CK8e promoter driving the production of the Z-disk fragment, endogenous translation of full-length nebulin gets overwhelmed, resulting in a gradual replacement of full-length nebulin by the Z-disk fragment. Only in *Neb* cKO muscles, where nebulin is absent and binding sites are readily available, does the Z-disk fragment incorporate with no adverse effects.

Recently, a dominant-negative nebulin mutation was reported in patients that strongly resembles the phenotypes observed in this study [76]. In brief, the patient’s muscles produced a mix of full-length and truncated nebulin, resulting in a slowly progressive myopathy. While it has been shown that mice lacking one nebulin allele have minimal to no overt phenotypes [77, 78], the presence of truncated nebulin appears to have a more deleterious effect. Thus, in a healthy sarcomere where thin filaments are fully decorated with nebulin, the introduction of a nebulin fragment may cause displacement of the full-length protein. Additional studies must also be performed to account for the fact that patients with nemaline myopathy often retain some full-length nebulin [3, 35, 79, 80]. But while an even lower dosage may be warranted, the 1× dosage group in *Neb* cKO mice already expresses a physiologically relevant expression of the Z-disk fragment (Fig. 2e) with minimal functional changes. Lower dosages are likely to result in the loss of the structural benefits, discussed below.

### Effects of the Z-disk fragment on sarcomere structure

Immunofluorescence studies showed that the Z-disk fragment expressed in CTRL and *Neb* cKO mice localized specifically to the Z-disks of the sarcomeres (Fig. 3). These data support the conclusion that the Z-disk fragment is being integrated thoroughly into the skeletal muscle sarcomere. Additionally, its localization would properly support nebulin-deficient *Neb* cKO sarcomeres as was initially expected and, at the same time, compete with full-length nebulin in control sarcomeres.

In AAV-treated CTRL muscles, protein aggregates (rod bodies) and horizontal displacement of the myofibrils were observed (Fig. 4a, insert). These phenotypes are likely caused by the loss of full-length nebulin, creating an environment more like that of nemaline myopathy. Conversely, AAV-treated *Neb* cKO muscles were found to have significantly smaller aggregates (Fig. 4b, left). This implies that the development of structural disorganization associated with nemaline myopathy is being delayed. Because the Z-disk portion of nebulin, specifically the final two domains, regulates the structure of the Z-disk [31], we also analyzed the changes in Z-disk width in these muscles. While the AAV-treated CTRL muscles experienced a significant widening of the Z-disks, AAV-treated *Neb* cKO muscles experienced a slight but significant decrease in the Z-disk widths (Fig. 5a, b). Previous studies on nemaline myopathy have reported Z-disk widening as a change associated with the onset of the disease [32, 33]. It is promising to see that the introduction of nebulin’s Z-disk into *Neb* cKO muscle may be slowing down phenotype development.

Because changes in Z-disk width often indicate changes in fiber-type composition [81], myosin heavy chain (MHC) was studied. It is known that MHC composition shifts toward slower fiber types as muscles lose nebulin [36]. In this study, AAV-treated *Neb* cKO mice were found to contain more type IIB (fast) MHC and less type I (slow) MHC (Fig. 6c). Simultaneously, CTRL mice lose type IIB MHC and gain more type IIA/X (slow) MHC (Fig. 6b). Overall, this data suggests that the expression of this Z-disk fragment in nebulin-deficient mice attenuates changes in fiber-type composition within the muscle.

### Impact on force production and contractile kinetics

A major goal in the treatment of nemaline myopathy is the restoration of force production. To test the effect of the AAV treatment on force production, in vitro whole-muscle mechanical studies were used to quantify force production. Though subtle changes were found in the force-frequency response of the *Neb* cKO mice, a small force increase was only detected at the 1× AAV dose (Fig. 7a, left; Additional file 1: Figure S4A, B). In contrast, CTRL mice experienced a significant loss of maximal tetanic force (Fig. 7a, b), likely due to the reduction of full-length nebulin and loss of its essential functions in muscle contraction. And other than having a significant effect on muscle relaxation (Additional file 1: Figure S5B, right), the Z-disk fragment was found to have a neutral effect on nebulin-deficient muscles. This implies that the Z-disk fragment assists primarily in the stabilization of the Z-disks. *Neb* cKO mice have shortened thin filament lengths and decreased actomyosin interactions [32, 33, 36, 37, 82, 83], which do not appear to be positively impacted by the presence of the Z-disk fragment. As nebulin is a massive, multi-functional protein, treatment of nemaline myopathy might not be attainable to a sufficient degree with only a Z-disk fragment and additional studies will be needed first. It is likely that additional nebulin fragments must be used to more fully reverse the phenotype.

Changes in the Z-disk widths are inherently tied to changes in MHC isoforms, with widening associated with slower fiber types [84, 85]. Differences in contractility can also be observed in the fatigue response of fast- and slow-twitch muscles. Slow-twitch muscles like the soleus exhibit a gradual decline in force production when subjected to a fatigue protocol, revealing its resistance to fatigue [56]. Nebulin-deficient EDL muscles behave in a similar fashion (Fig. 8a). This is likely caused by the drastic shift toward slow MHC isoforms in *Neb* cKO EDL muscles [36]. That shift may be a physiological response to an increased ATP tensions cost, as slower fiber types are accompanied by increased mitochondrial count and improved exercise tolerance [81, 86]. While this shift in MHC isoform distribution has been slowed due to the AAV treatment (Fig. 6, Additional file 1: Figure S3), the *Neb* cKO EDL muscle is still significantly different than a CTRL muscle. Thus, the fatigue response in AAV-treated *Neb* cKO muscles does not resemble that of CTRL muscles. Despite this, AAV-treated *Neb* cKO muscles exhibit higher fatigue resistance (Fig. 8b, c). This suggests that along with slowing the MHC isoform shift, the AAV treatment has improved energy consumption and bioenergetics of nebulin-deficient muscles. Further studies into ATP usage and mitochondrial distribution are needed to extend this observation.

### Changes in regulatory proteins associated with nemaline myopathy

We also studied the recently-discovered proteins associated with the development of nemaline myopathy, KLHL41 and NRAP. Kelch-like family member 41, KLHL41, was selected for its role in nebulin stabilization as well as possible ubiquitination processes in nemaline myopathy [19, 64]. Both overexpression and loss of KLHL41 have been shown to cause nemaline myopathy-like phenotypes [19, 87], implying that its regulation is critical to sarcomeric structure. KLHL41 was also reported to regulate nebulin-related anchoring protein, NRAP [65]. Interestingly, when this protein is removed in KLHL41-deficient muscle, the myopathy phenotypes are reversed [65]. Quantification of the expression of these two proteins found that CTRL muscles produce more of both proteins in AAV-treated muscles (Fig. 9b, c). This might reflect the AAV-treated CTRL muscle’s gradual progression toward myopathy. Conversely, AAV-treated *Neb* cKO muscles only experienced an increase in KLHL41 expression in TC muscles (Fig. 9b). This may indicate that, while the expression of the Z-disk fragment in nebulin-deficient muscle has slowed the progression of structural changes in nemaline myopathy, it has not been sufficient to alter some regulatory proteins. The high expression of KLHL41 and NRAP would further hinder the organization of mature thin filaments within the sarcomeres, contributing to the disease phenotype. Again, we find that the introduction of only the Z-disk fragment of nebulin is insufficient to rescue this phenotype.

Through additional studies of Z-disk-associated proteins (Additional file 1: Figure S8 and S9), we did however identify four proteins that may be reflective of pathology. CSRP3, myozenin-1, myozenin-2, and the long isoform of cypher all showed significant (or trending) treatment effects via a repeated-measure two-way ANOVA (Additional file 1: Figure S9B, G-I). CSRP3 (also referred to as muscle LIM protein) is a critical regulator of sarcomeric development within striated muscle, with a prominent role in myogenesis and Z-disk regulation [88]. Myozenin-1 and myozenin-2 interact with the cypher isoforms in complex within the Z-disk to regulate its structure [89–91]. Additionally, myozenin-1 and myozenin-2 play a role in fiber-type switching. The reduction in protein expression may also indicate a slowing of pathology, wherein overexpression was tied to the development of a nemaline myopathy phenotype. Interestingly, each of these proteins also plays a role in calcineurin regulation [88–91]. Future work is needed to address the importance of changes in Z-disk associated proteins.

## Conclusion

The key finding in this study is that nebulin-deficient muscle can readily incorporate the Z-disk region of nebulin. Additionally, there are no significant negative effects on structure and force production in *Neb* cKO mice. Supplementing these mice with the Z-disk fragment appears to slow the progression of nemaline myopathy but does not reverse it. Removal of nebulin’s C-terminal domains does not affect thin filaments [31] and the present study shows that stabilizing the Z-disks in *Neb* cKO mice does not increase shortened thin filament lengths. It may be possible that the introduction of additional nebulin fragments in addition to the Z-disk fragment could serve to stabilize the thin filaments and augment crossbridge interaction. For instance, expressing nebulin’s super-repeats in conjunction with the Z-disk fragment may serve to repair thin filament function and improve force production.

We also show that a notable complication when using nebulin fragments in nebulin-deficient muscle is its interaction with full-length nebulin. This is because the C-terminus of nebulin has a high affinity to the Z-disk of the sarcomeres and a fragment containing this region can displace full-length nebulin. From a clinical standpoint, patients still retain partial expression of full-length nebulin [3, 35, 79, 80]. Should treatments similar to the one used in this study be considered, additional studies into the effect of treatment on residual full-length nebulin expression need to be conducted first. In the end, full-length nebulin is too large to express through current technologies. Here, we have taken a first step into replicating nebulin’s presence by integrating key domains back into nebulin-deficient sarcomeres. As the current treatment is well-tolerated, the next step will be to express additional regions of nebulin to determine if higher degrees of structural and functional recovery are possible.

## Supplementary information

**Additional file 1: Figure S1.** Effect of AAV treatment on muscle weights (TC and EDL) and physiological cross-sectional area (PCSA) in EDL muscle. A) Vehicle-treated and AAV-treated, CTRL and *Neb* cKO TC weights in the 1x (left) and 3x (right) dosage groups. Paired t-test revealed a small but significant decrease in the *Neb* cKO TC weights of the 1x dosage group. This difference was not replicated in the 3x dosage group. B) Vehicle-treated and AAV-treated, CTRL and *Neb* cKO EDL weights in the 1x (left) and 3x (right) dosage groups. No significant differences were found using paired t-tests between AAV-treated EDLs and their contralateral, vehicle-treated muscles. C) PCSAs of the EDL muscles in both 1x (left) and 3x (right) dosage groups (see Methods for details). AAV treatment had no significant effect on the cross-sectional areas. (1x: *n* = 9,12 mice; 3x: *n* = 4,9 mice). **Figure S2.** Localization of the Z-disk AAV construct in the 3x AAV dosage group. **Figure S3.** High magnification TEM images of Z-disk structure in *Neb* cKO TCs. Even at high 43,000x magnification, the differences in Z-disk widths are difficult to discern. Z-disks were precisely measured using the full-width, half-max values of a gaussian fit of the gray values. **Figure S4.** Quantification of MHC composition in 3x dosage treatment group. A) Quantification of MHC composition in vehicle-treated and AAV-treated CTRL TCs and EDLs (3x dosage). Paired t-tests reveal a significant increase in Type IIA/X MHC and a significant decrease in Type IIB MHC in both muscle types. (*n* = 4 mice). B) Quantification of MHC composition in vehicle-treated and AAV-treated *Neb* cKO TCs and EDLs (3x dosage). Paired t-tests reveal a significant increase in Type IIB MHC and a significant decrease in Type I MHC. (*n* = 8 mice). **Table S1.** Specific force results at each stimulation frequency were compared at the 1x dose (A) and 3x dose (B). (1x: *n* = 9,11 mice; 3x: *n* = 4,9 mice). **Figure S5.** Normalized force-frequency curves. A) Normalized force-frequency (FF) curves at 1x (left) and 3x (right) dosages. The *Neb* cKO curves are left-shifted from CTRL curves. No consistent AAV-effect is seen. (Forces at each stimulation frequency were divided by the maximal tetanic force). B) Quantification of frequency that results in half-maximal force at 1x (left) and 3x (right) dosages. A significant reduction in the ‘half-max frequency’ in *Neb* cKO mice treated with the 1x dosage (paired t-test) occurs. This decrease is lost in the 3x dosage group and no effect is present in the CTRL group. (Paired t-tests were used in this analysis). (1x: *n* = 9,11 mice; 3x: *n* = 4,9 mice). **Figure S6.** Analysis of contraction kinetics in both 1x and 3x dosage groups. A) Time to max force during a twitch (left) and a 200 Hz tetanus (right). *Neb* cKO mice have a significantly longer time to maximal twitch force in the 3x dosage group and a significantly longer time to max tetanic force in the 1x dosage group. B) Half-relaxation time after the twitch (left) and the 200 Hz tetanus (right). While *Neb* cKO mice have a significantly faster relaxation time in the 3x dosage group, none of the other comparisons had a change in twitch relaxation time. Comparatively, all groups except for the *Neb* cKO mice in the 3x dosage group had significantly longer relaxation times following a maximal tetanus. (Paired t-tests were used in these analyses. 1x: *n* = 9,11 mice; 3x: *n* = 4,9 mice). **Figure S7.** Expression of nebulin regulatory proteins in 3x dosage group. (*n* = 4,9 mice). **Figure S8.** Representative blots pertaining to different components of the Z-disks. (*n* = 3–5 mice). **Figure S9.** Analyses of Z-disk protein expression. (*n* = 3–5 mice).

## Data Availability

All data generated or analyzed are included in this published article and its Additional information files.

## Abbreviations

AAV: Adeno-associated virus
cKO: Conditional knockout
EDL: Extensor digitorum longus
KLHL41: Kelch-like protein 41 (also known as Kelch repeat and BTB Domain Containing 10, KBTBD10)
MHC: Myosin heavy chain
NRAP: Nebulin-related anchoring protein
PCSA: Physiological cross-sectional area
TC: Tibialis cranialis (also referred to as tibialis anterior)
