# Correction to: Expressing a Z-disk nebulin fragment innebulin-deficient mouse muscle: effects on muscle structure and function

**DOI:** 10.1186/s13395-020-00223-8

**Published:** 2020-04-20

**Authors:** Frank Li, Justin Kolb, Julie Crudele, Paola Tonino, Zaynab Hourani, John E. Smith, Jeffrey S. Chamberlain, Henk Granzier

**Affiliations:** 1 Department of Cellular and Molecular Medicine, University of Arizona, Tucson, AZ 85721 USA; 2 Department of Neurology, University of Washington, Seattle, WA 98109-8055 USA; 3 Medical Research Building, RM 325, 1656 E Mabel St, Tucson, AZ 85721 USA

**Correction to: Skeletal Muscle (2020)10:2**

**https://doi.org/10.1186/s13395-019-0219-9**

Following the publication of this paper [1], it was brought to the authors’ attention that one of the contributing authors was left off of the paper. The authors apologize for the unfortunate oversight. In this correction paper, they have included Dr. Paola Tonino in the author list section.
