# Supplementary material for: Expressing a Z-disk nebulin fragment in nebulin-deficient mouse muscle: effects on muscle structure and function
Source: Skelet Muscle. 2020 Jan 28;10:2. doi: 10.1186/s13395-019-0219-9 (PMC6986074; doi:10.1186/s13395-019-0219-9)
Supplement: Supplementary file 1 — Additional file 1: Figure S1. Effect of AAV treatment on muscle weights (TC and EDL) and physiological cross-sectional area (PCSA) in EDL muscle. A) Vehicle-treated and AAV-treated, CTRL and Neb cKO TC weights in the 1x (left) and 3x (right) dosage groups. Paired t-test revealed a small but significant decrease in the Neb cKO TC weights of the 1x dosage group. This difference was not replicated in the 3x dosage group. B) Vehicle-treated and AAV-treated, CTRL and Neb cKO EDL weights in the 1x (left) and 3x (right) dosage groups. No significant differences were found using paired t-tests between AAV-treated EDLs and their contralateral, vehicle-treated muscles. C) PCSAs of the EDL muscles in both 1x (left) and 3x (right) dosage groups (see Methods for details). AAV treatment had no significant effect on the cross-sectional areas. (1x: n = 9,12 mice; 3x: n = 4,9 mice). Figure S2. Localization of the Z-disk AAV construct in the 3x AAV dosage group. Figure S3. High magnification TEM images of Z-disk structure in Neb cKO TCs. Even at high 43,000x magnification, the differences in Z-disk widths are difficult to discern. Z-disks were precisely measured using the full-width, half-max values of a gaussian fit of the gray values. Figure S4. Quantification of MHC composition in 3x dosage treatment group. A) Quantification of MHC composition in vehicle-treated and AAV-treated CTRL TCs and EDLs (3x dosage). Paired t-tests reveal a significant increase in Type IIA/X MHC and a significant decrease in Type IIB MHC in both muscle types. (n = 4 mice). B) Quantification of MHC composition in vehicle-treated and AAV-treated Neb cKO TCs and EDLs (3x dosage). Paired t-tests reveal a significant increase in Type IIB MHC and a significant decrease in Type I MHC. (n = 8 mice). Table S1. Specific force results at each stimulation frequency were compared at the 1x dose (A) and 3x dose (B). (1x: n = 9,11 mice; 3x: n = 4,9 mice). Figure S5. Normalized force-frequency curves. A) Normalized force- [file 13395_2019_219_MOESM1_ESM.pdf]

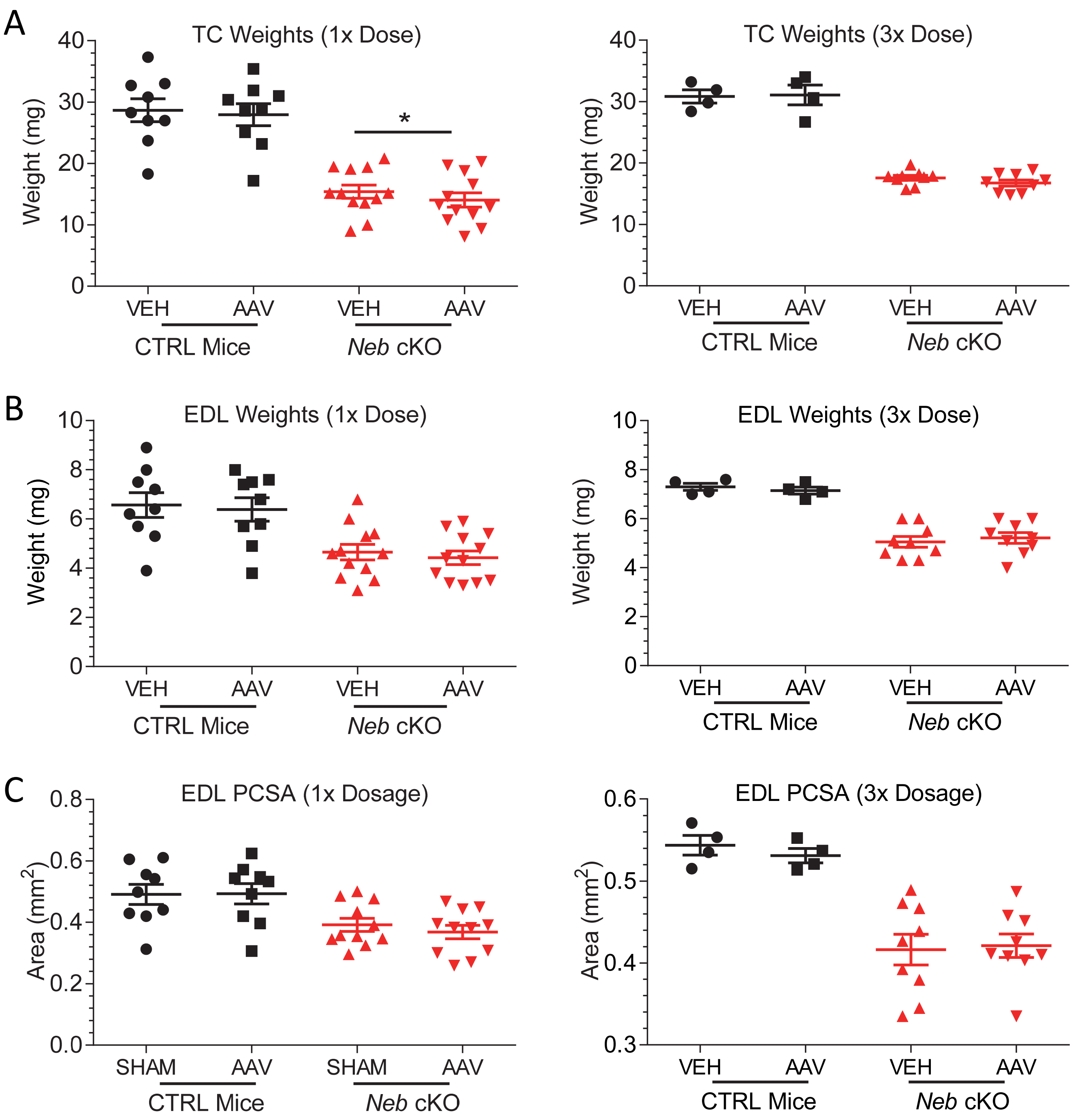

Supplemental Figure 1. Effect of AAV treatment on muscle weights (TC and EDL) and physiological cross-sectional area (PCSA) in EDL muscle.

A) Vehicle-treated and AAV-treated, CTRL and *Neb* cKO TC weights in the 1x (left) and 3x (right) dosage groups. Paired t-test revealed a small but significant decrease in the *Neb* cKO TC weights of the 1x dosage group. This difference was not replicated in the 3x dosage group.

B) Vehicle-treated and AAV-treated, CTRL and *Neb* cKO EDL weights in the 1x (left) and 3x (right) dosage groups. No significant differences were found using paired t-tests between AAV-treated EDLs and their contralateral, vehicle-treated muscles.

C) PCSAs of the EDL muscles in both 1x (left) and 3x (right) dosage groups (see Methods for details). AAV treatment had no significant effect on the cross-sectional areas.

(1x: n=9,12; 3x: n=4,9)

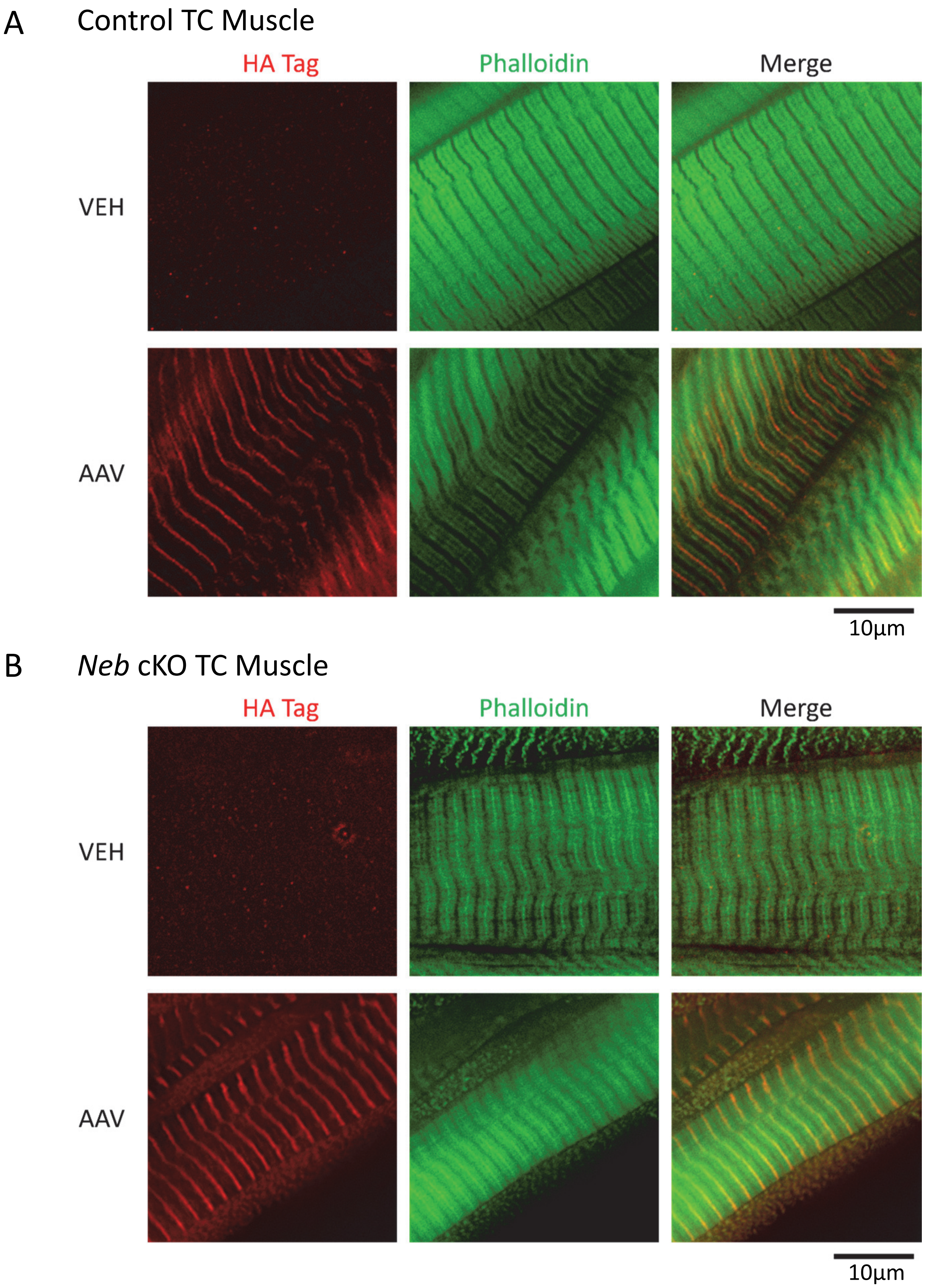

Supplemental Figure 2. Localization of the Z-disk AAV construct in the 3x AAV dosage group.

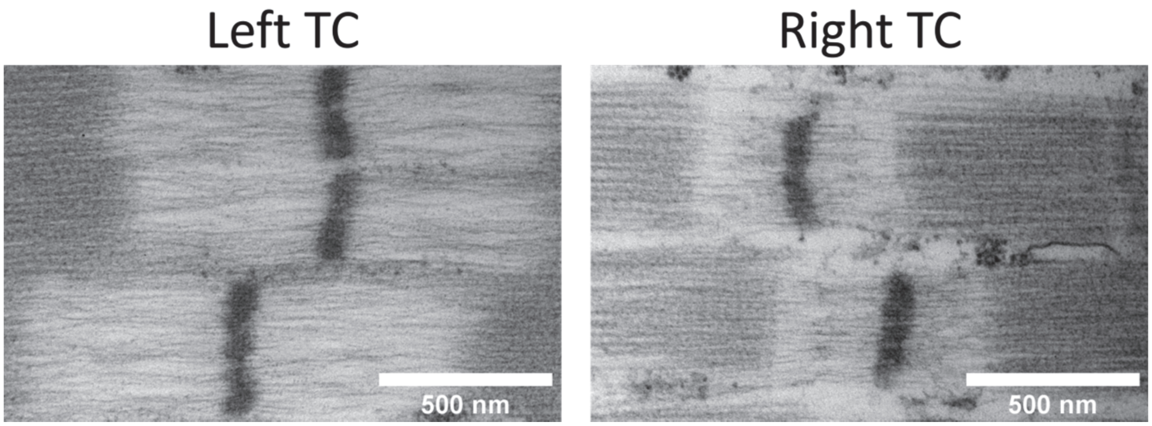

Supplemental Figure 3. High magnification TEM images of Z-disk structure in *Neb* cKO TCs. Even at high 43,000x magnification, the differences in Z-disk widths between left (Vehice) and right (AAV) are difficult to discern. Z-disks were precisely measured using the full-width, half-max values of a gaussian fit of the gray values.

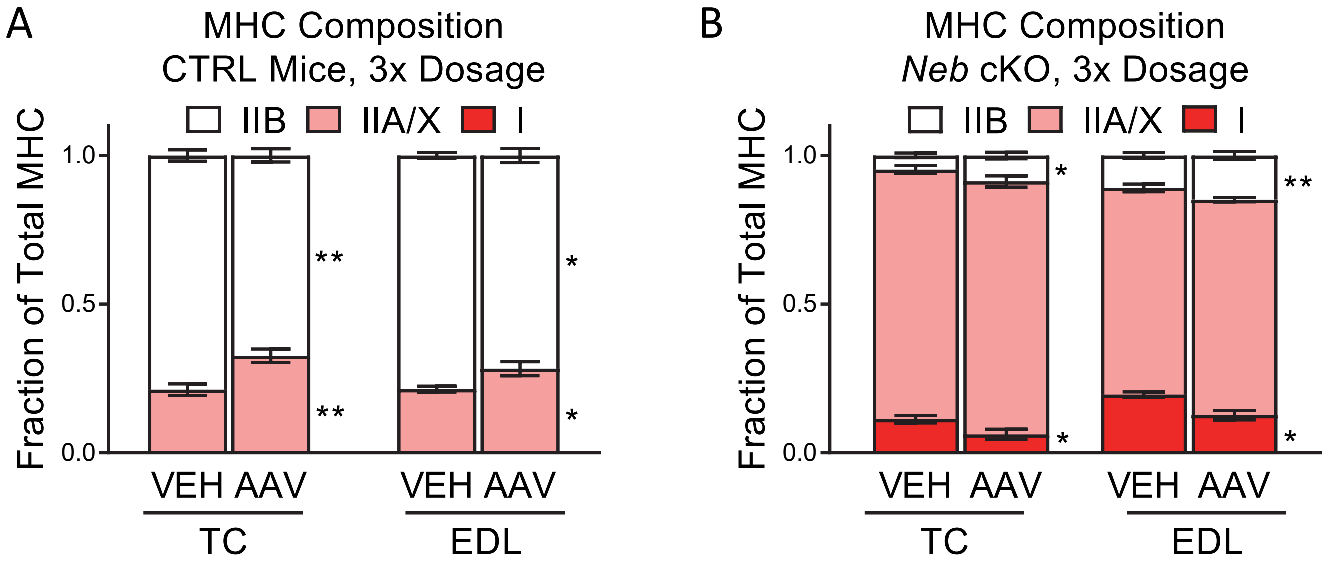

Supplemental Figure 4. Quantification of MHC composition in 3x dosage treatment group.

A) Quantification of MHC composition in vehicle-treated and AAV-treated CTRL TCs and EDLs (3x dosage). Paired t-tests reveal a significant increase in Type IIA/X MHC and a significant decrease in Type IIB MHC in both muscle types. (n=4)

B) Quantification of MHC composition in vehicle-treated and AAV-treated *Neb* cKO TCs and EDLs (3x dosage). Paired t-tests reveal a significant increase in Type IIB MHC and a significant decrease in Type I MHC. (n=8)

A

1x Dosage	CTRL Mice (AAV vs VEH)		*Neb* cKO (AAV vs VEH)	
Frequency	Significance	P-Value	Significance	P-Value
1	ns	0.9941	ns	>0.9999
5	ns	0.9964	ns	>0.9999
10	ns	0.9933	ns	>0.9999
20	ns	0.9680	ns	>0.9999
40	ns	0.3168	ns	0.1496
60	**	0.0026	ns	0.1748
80	***	0.0002	ns	0.1612
100	****	<0.0001	ns	0.1738
150	****	<0.0001	ns	0.2284
200	****	<0.0001	ns	0.3077

B

3x Dosage	CTRL Mice (AAV vs VEH)		*Neb* cKO (AAV vs VEH)	
Frequency	Significance	P-Value	Significance	P-Value
1	ns	>0.9999	ns	>0.9999
5	ns	>0.9999	ns	>0.9999
10	ns	>0.9999	ns	>0.9999
20	ns	>0.9999	ns	>0.9999
40	ns	0.9891	ns	0.9451
60	ns	0.7041	ns	0.9585
80	ns	0.3504	ns	0.9161
100	ns	0.1661	ns	0.8707
150	*	0.0158	ns	0.9664
200	**	0.0044	ns	0.9747

Supplemental Table 1. Specific force results at each stimulation frequency were compared at the 1x dose (A) and 3x dose (B). (1x: n=9,11; 3x: n=4,9)

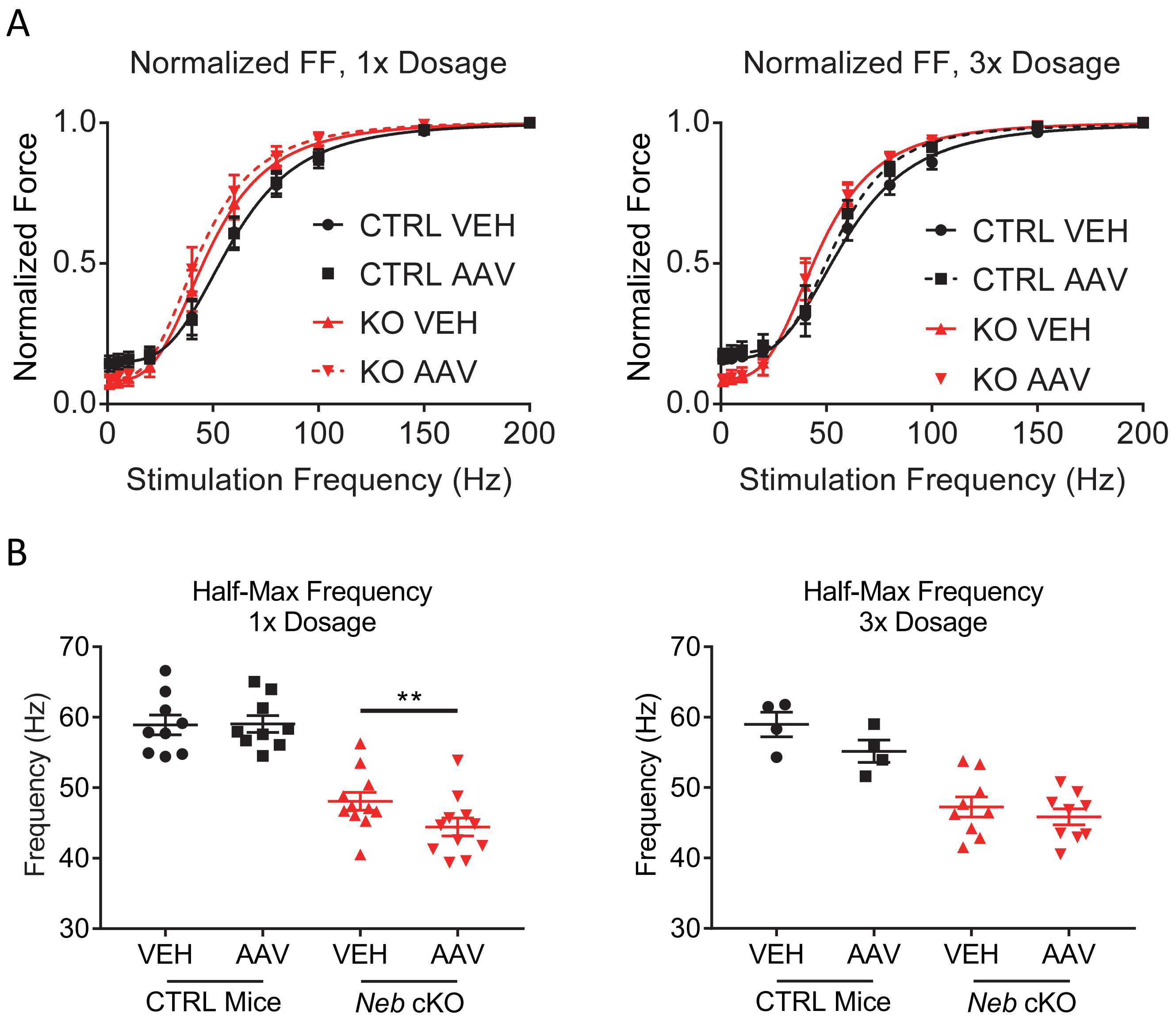

Supplemental Figure 5. Normalized force-frequency curves.

A) Normalized force-frequency (FF) curves at 1x (left) and 3x (right) dosages. The *Neb* cKO curves are left-shifted from CTRL curves. No consistent AAV-effect is seen. (Forces at each stimulation frequency were divided by the maximal tetanic force)

B) Quantification of frequency that results in half-maximal force at 1x (left) and 3x (right) dosages. A significant reduction in the 'half-max frequency' in *Neb* cKO mice treated with the 1x dosage (paired t-test) occurs. This decrease is lost in the 3x dosage group and no effect is present in the CTRL group. (Paired t-tests were used in this analysis)

(1x: n=9,11; 3x: n=4,9)

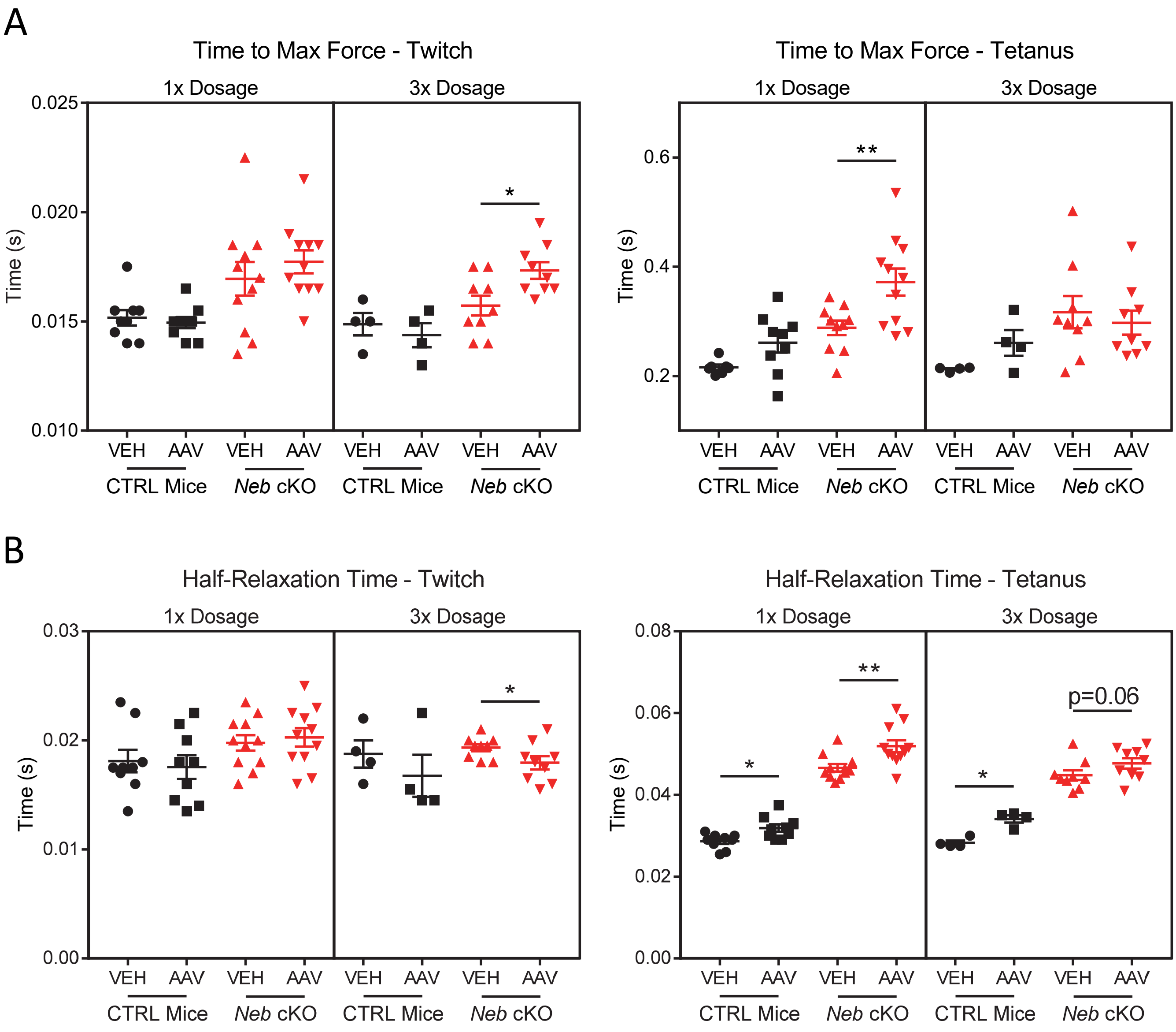

Supplemental Figure 6. Analysis of contraction kinetics in both 1x and 3x dosage groups.
A) Time to max force during a twitch (left) and a 200Hz tetanus (right). *Neb* cKO mice have a significantly longer time to maximal twitch force in the 3x dosage group and a significantly longer time to max tetanic force in the 1x dosage group.

B) Half-relaxation time after the twitch (left) and the 200Hz tetanus (right). While *Neb* cKO mice have a significantly faster relaxation time in the 3x dosage group, none of the other comparisons had a change in twitch relaxation time. Comparatively, all groups except for the *Neb* cKO mice in the 3x dosage group had significantly longer relaxation times following a maximal tetanus.

(Paired t-tests were used in these analyses. 1x: n=9,11; 3x: n=4,9)

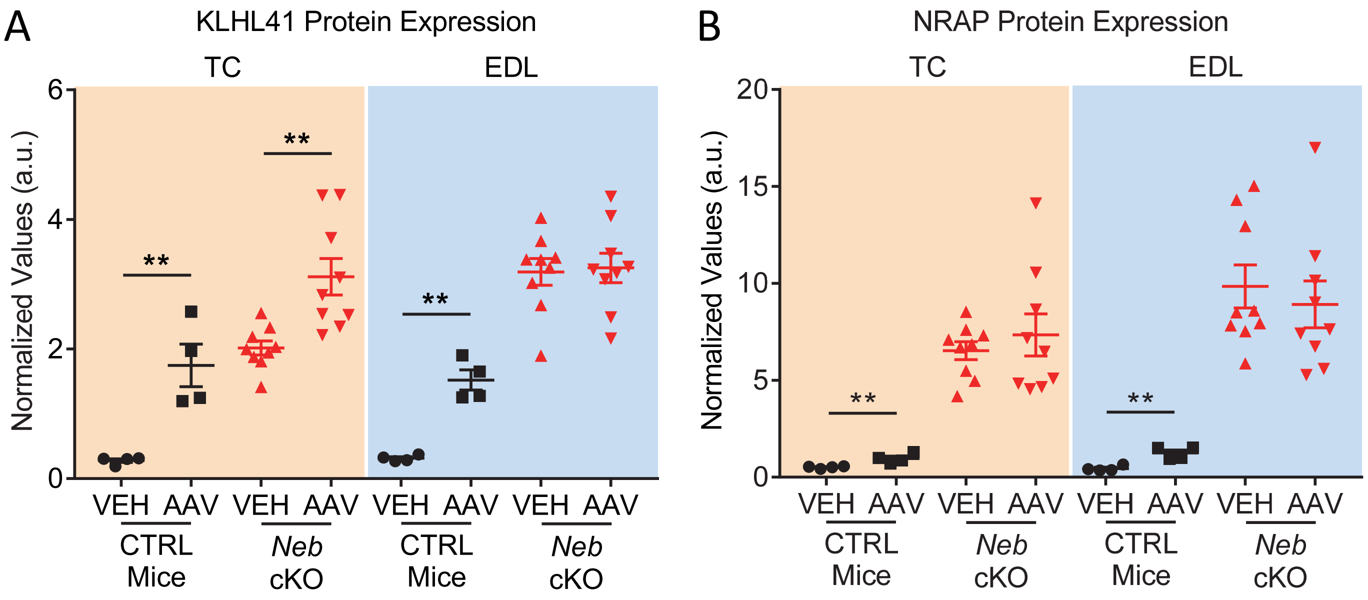

Supplemental Figure 7. Expression of nebulin regulatory proteins in 3x dosage group. (n=4,9)

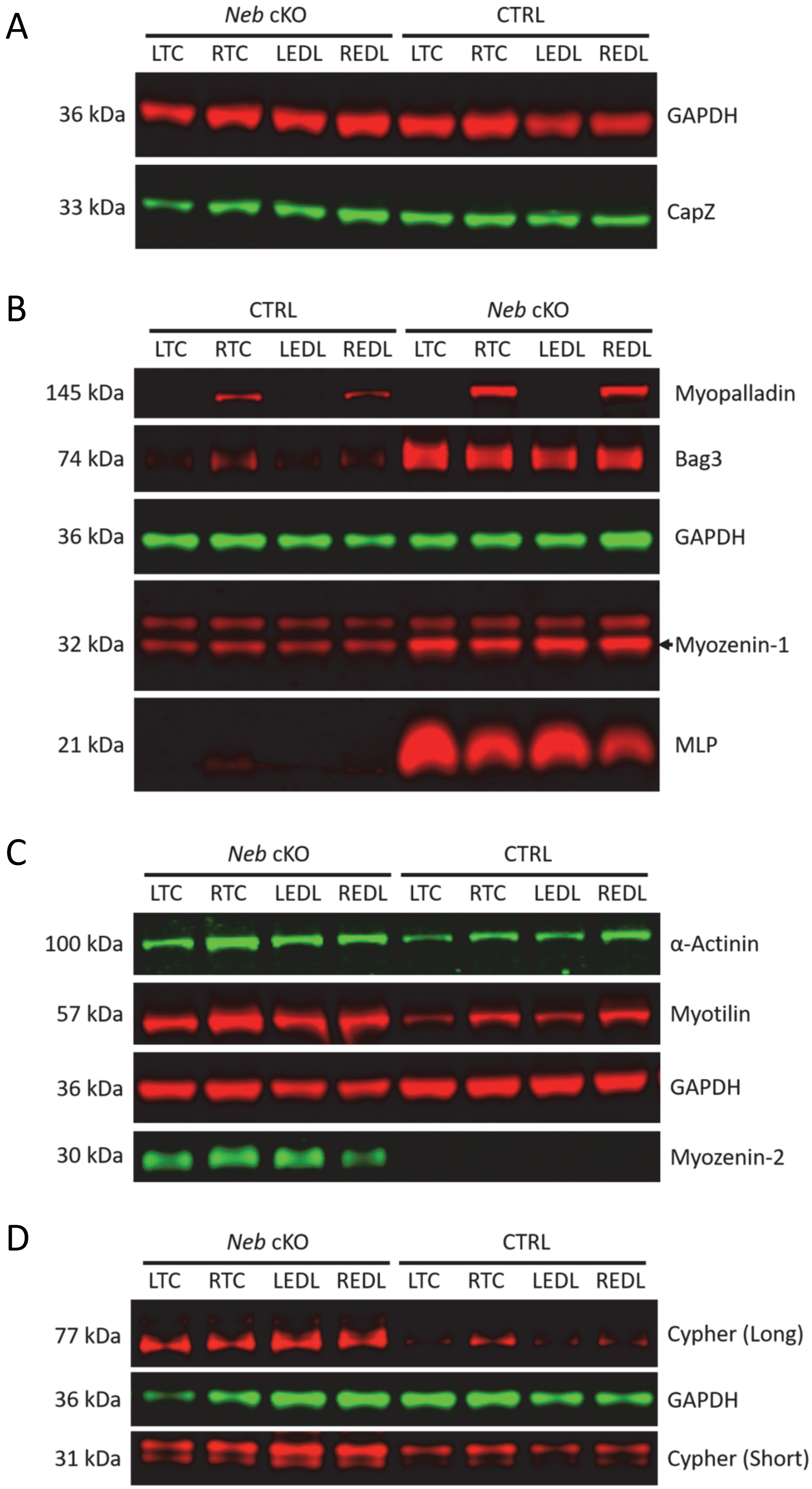

Supplemental Figure 8. Representative blots pertaining to different components of the Z-disks. (n=3-5)

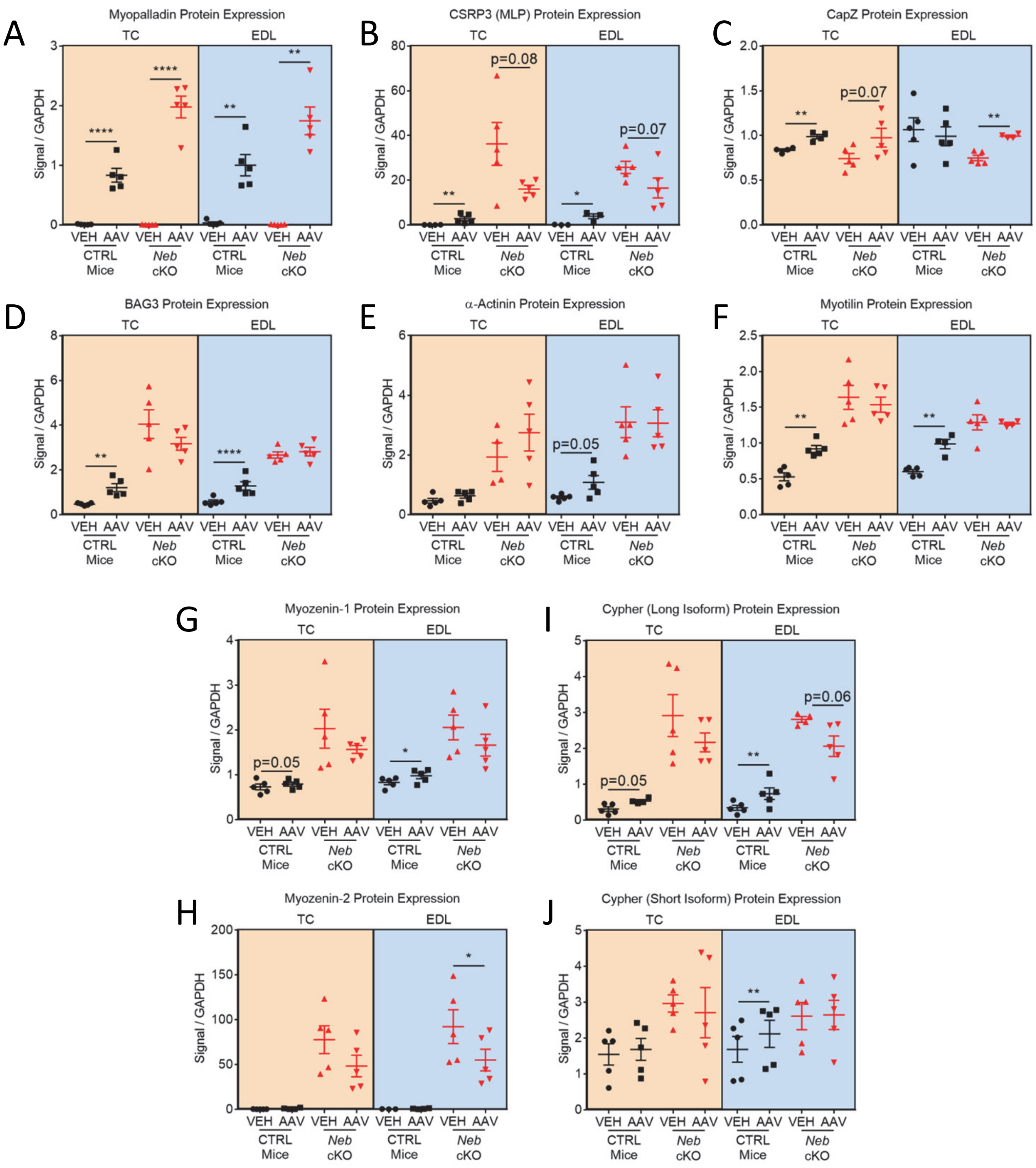

Supplemental Figure 9. Analyses of Z-disk protein expression.
